# Controlled Chaos of Polymorphic Mucins in a Metazoan Parasite *(Schistosoma mansoni)* Interacting with Its Invertebrate Host *(Biomphalaria glabrata)*

**DOI:** 10.1371/journal.pntd.0000330

**Published:** 2008-11-11

**Authors:** Emmanuel Roger, Christoph Grunau, Raymond J. Pierce, Hirohisa Hirai, Benjamin Gourbal, Richard Galinier, Rémi Emans, Italo M. Cesari, Céline Cosseau, Guillaume Mitta

**Affiliations:** 1 Parasitologie Fonctionnelle et Evolutive, UMR 5244, CNRS Université de Perpignan, Perpignan, France; 2 Inserm, U 547, Université Lille 2, Institut Pasteur de Lille, IFR 142, Lille, France; 3 Primate Research Institute, Kyoto University, Inuyama, Aichi, Japan; 4 Laboratoire de Parasitologie, Faculté de Médecine, U.L.B CP 616, Bruxelles, Belgique; George Washington University Medical Center, United States of America

## Abstract

Invertebrates were long thought to possess only a simple, effective and hence non-adaptive defence system against microbial and parasitic attacks. However, recent studies have shown that invertebrate immunity also relies on immune receptors that diversify (e.g. in echinoderms, insects and mollusks (*Biomphalaria glabrata*)). Apparently, individual or population-based polymorphism-generating mechanisms exists that permit the survival of invertebrate species exposed to parasites. Consequently, the generally accepted arms race hypothesis predicts that molecular diversity and polymorphism also exist in parasites of invertebrates. We investigated the diversity and polymorphism of parasite molecules (*Schistosoma mansoni* Polymorphic Mucins, *Sm*PoMucs) that are key factors for the compatibility of schistosomes interacting with their host, the mollusc *Biomphalaria glabrata*. We have elucidated the complex cascade of mechanisms acting both at the genomic level and during expression that confer polymorphism to *Sm*PoMuc. We show that *Sm*PoMuc is coded by a multi-gene family whose members frequently recombine. We show that these genes are transcribed in an individual-specific manner, and that for each gene, multiple splice variants exist. Finally, we reveal the impact of this polymorphism on the *Sm*PoMuc glycosylation status. Our data support the view that *S. mansoni* has evolved a complex hierarchical system that efficiently generates a high degree of polymorphism—a “controlled chaos”—based on a relatively low number of genes. This contrasts with protozoan parasites that generate antigenic variation from large sets of genes such as *Trypanosoma cruzi*, *Trypanosoma brucei* and *Plasmodium falciparum*. Our data support the view that the interaction between parasites and their invertebrate hosts are far more complex than previously thought. While most studies in this matter have focused on invertebrate host diversification, we clearly show that diversifying mechanisms also exist on the parasite side of the interaction. Our findings shed new light on how and why invertebrate immunity develops.

## Author Summary

Contrary to the traditional view that immunity in invertebrates is limited to non-specific mechanisms, recent studies have shown that they have diverse, specific immune receptors. An example is provided by the FREPs of the mollusk *Biomphalaria glabrata*, polymorphic members of the immunoglobulin superfamily. This capacity for an individual or population-based polymorphic immune response raises the question of whether a corresponding polymorphism exists in parasites of invertebrates, as would be expected in an “arms race” between host and parasite. We have indeed identified such polymorphic molecules in *Schistosoma mansoni*, a flatworm parasite of *B. glabrata*, by comparing two strains of schistosome that are respectively compatible and incompatible with the same mollusk host strain. However, in contrast to antigenic variation in protozoan parasites that is based on an extensive gene repertoire, we show here that a high level of polymorphism in these *S. mansoni* polymorphic mucins (*Sm*PoMucs) is generated from a low number of genes by a complex cascade of mechanisms, a “controlled chaos”.

## Introduction

The comprehension of host-parasite interactions represents a major challenge in evolutionary biology. Parasites are responsible for substantial deleterious effects on their hosts, and therefore represent a major driving force for their evolution. In parallel, parasites have to cope with the evolving host-defence mechanisms, i.e. they must co-evolve with their host to avoid elimination. This adaptation of the Red Queen hypothesis [1] to host-parasite systems predicts that an arms race takes place in which both host and parasite develop mechanisms that generate diversity and polymorphism of molecules that play key roles in the host-parasite interplay [2].

In vertebrate hosts, the most striking example is the exceptional diversity of antigen-specific receptors of the adaptive immune system of jawed vertebrates. This system depends on somatic gene rearrangement and hypermutation [3]–[5]. For the pathogen counterparts, a variety of mechanisms permitting evasion of the host's immune response exist in pathogenic bacteria and viruses [6] and antigenic variation is a widespread strategy for most of the eukaryotic parasites [7]. In the case of invertebrate hosts and their parasites, the picture is believed to be completely different since the prevailing view is that invertebrates have no acquired adaptive immunity, and that their immune system is innate and “non-specific”. The detection of parasites by invertebrates was thought to rely exclusively on invariable germline-encoded Pattern Recognition Receptors (PRRs) that recognize pathogen-associated molecular patterns (PAMPs) [8]. Nevertheless, recent studies have shaken this paradigm by providing evidence for novel and diverse immune receptor sequences in protochordates (*Amphioxus*; [9]), in echinoderms (sea urchin; [10]), insects (*Drosophila melanogaster and Anopheles gambiae*; [11],[12]) and mollusks (*Biomphalaria glabrata*; [13]). These results suggest the existence of individual or population-based polymorphism permitting the survival of individuals or species confronted with parasites. These recent observations raise the question of whether diversity and polymorphism also exist in key compatibility molecules expressed by parasites (or parasite stages in the case of multi-host parasites) interacting with invertebrate hosts, whether these molecules are subject to variation and whether molecular polymorphism is at the core of interaction with the invertebrate host immune system.

To address these questions, we focused our study on a host-parasite model where the co-evolutionary dynamics is accessible: a model in which only some particular host and parasite phenotypes are compatible. We analyzed the interaction between *Schistosoma mansoni*, the agent of human intestinal schistosomiasis [14] and its invertebrate intermediate host, the gastropod mollusk *B. glabrata*. In this interaction, compatibility polymorphism occurs [15], i.e. in natural populations some snail/schistosome combinations are compatible and others are not. We hypothesized that this compatibility polymorphism is dependent on diversification mechanisms that act on key molecules such as the PRRs of the immunoglobulin superfamily (IgSF) characterized in *B. glabrata* (FREPs: Fibrinogen Related Proteins, [15]) and parasite antigens. The *FREPs* genes encode lectin-like hemolymph polypeptides that can precipitate soluble antigens derived from trematodes [16]. FREPs proteins consist of one or two amino-terminal IgSF domains and a carboxyl-terminal fibrinogen domain. These molecules undergo mutations and recombinatorial processes that lead to diversification [13]. According to the arms race hypothesis, polymorphic molecular variants expressed by schistosome larvae in intermediate hosts could explain the observed compatibility polymorphism. While some parasites like *Plasmodium falciparum* or *Trypanosoma sp.* have developed a rich repertoire of mechanisms to generate polymorphic variants, the system that generates diversity in *S. mansoni* is so far unknown. We have previously shown by a comparative proteomics approach [17] that the principal difference between compatible and incompatible strains of *S. mansoni* is the presence of particular *Sm*PoMuc protein variants. We have described the principal characteristic of the coding sequence, gene expression patterns and protein localization of *Sm*PoMuc [18]. We have shown that these proteins are expressed and secreted by miracidia and sporocysts, i.e the larval stages that interact with the mollusk. In addition, we have described their high level of intra- and inter-strain polymorphism. Here, we elucidate the complex cascade of mechanisms that confers polymorphism to *Sm*PoMuc. We show that *Sm*PoMuc is coded by a multi-gene family. Genes are transcribed in individual-specific manner, and for each gene, multiple splice variants exist. The incidence of this polymorphism on *Sm*PoMuc glycosylation status is demonstrated. Our data support the view that *S. mansoni* has evolved a complex hierarchical system that efficiently generates highly polymorphic variants based on a relatively low number of genes.

## Materials and Methods

### Culture of *S. mansoni*

The compatible Brazilian (strain C) and incompatible Guadeloupean (strain IC) strains of *Schistosoma mansoni* were maintained in (i) *Biomphalaria glabrata* strains Bg.Bra and Bg.Gua, respectively and (ii) hamsters (*Mesocricetus auratus*) as described previously [19]. Adult worms and primary sporocysts (Sp1) were obtained as previously described [18]. Our laboratory has received the permit N° A 66040 for experiments on animals from both French Ministère de l'Agriculture et de la Pêche and French Ministère de l'Education Nationale de la Recherche et de la Technologie. Housing, breeding and animal care of the mice followed the ethical requirements of our country. The experimenter possesses the official certificate for animal experimentation delivered by both ministries (Décret n° 87–848 du 19 octobre 1987; number of the authorization 007083).

### Protein extraction, separation and detection

Two-D gel proteomic analysis was conducted according to procedures developed previously [17],[18]. Briefly, the total proteome of C and IC sporocysts originating from different hamster livers was extracted using 2D lysis buffer (8 M urea, 40 mM Tris, 4% CHAPS, 60 mM DTT). One hundred µg of protein were separated in the first dimension using 17 cm Ready Strip IPG Strips (Bio-Rad). Different pH gradients were used, a pH 3–10 non-linear gradient to have a broad overview of total protein distribution, and a pH 3–6 narrow-range gradient for increased resolution in the *Sm*PoMuc region. Isoelectrofocusing (IEF) was performed with voltage gradually increasing to 8000 V for 180 000 Vh at 20°C. Proteins were separated by 12% SDS-PAGE, visualized by silver staining [20] and the 2D gels were scanned using a densitometer (GS-800 Calibrated Densitometer, Bio-Rad).

### Chemical deglycosylation and western blotting

Chemical deglycosylation of *Sm*PoMuc proteins was performed using trifluoromethanesulfonic acid (TFMSA) according to a previously described procedure [21]. Briefly, 40 µg of each sample was treated with TFMSA and 1/2 volume of anisole and incubate on ice. TFMSA was neutralized with N-ethylmorpholine (NEM) and deglycosylated proteins were precipitated with acetone overnight at −20°C. Protein pellets were re-suspended in deionised water and Laemmli buffer and separated on a 12% SDS-PAGE. Proteins were transferred onto a nitrocellulose membrane (Hybond ECL, GE Healthcare) using semi-dry transfer (SemiPhor, Hoefer) and submitted to Western-Blot analysis.

The membrane was blocked with 5% non-fat dry milk in PBST (pH 7.4 PBS buffer containing 0.05% tween 20) overnight at 4°C and incubated with primary antibody (anti-*Sm*PoMuc IgG purified from rabbit) (1/200 in PBST) for 1.5 hours at room temperature. Incubation with secondary antibody (peroxidase conjugated, purified anti rabbit IgG) diluted 1/5000 was done in PBST for 1.5 hours at room temperature. After incubation with each antibody, the membrane was washed 3 times for 30 minutes with agitation in PBST. Detection was realized using ECL reagents (Pierce). The membrane was incubated with peroxidase substrate for 1 minute and exposed to X-ray film (GE Healthcare).

Confirmation of the removal of carbohydrate moieties was assessed by two procedures, Alcian blue staining and lectin blotting. For Alcian blue staining the SDS-PAGE gel was fixed in 7% acetic acid, stained (0.5% Alcian Blue) and de-stained in the same solution. Lectin blots were carried out after protein electrophoresis and transferred to a nitrocellulose membrane as previously and the membrane was incubated in PBST with a specific lectin peroxidase conjugate (concanavalin A for N-glycosylation, and jacalin for O-glycosylation). Chemiluminescent detection was as described previously.

### DNA and RNA extraction

Genomic DNA was extracted from *S. mansoni* adult worms using DNAzol Reagent (Invitrogen) according to the manufacturer's instructions. BAC clones containing *Sm*PoMuc genes (41B11, 62F12, 62J10, 47P6, 51E8 and 45D24) were grown up and BAC DNA preparations carried out as previously described [22].

Messenger RNA from individual sporocysts was isolated using the Dynabeads mRNA DIRECT Micro Kit (Invitrogen). Single Sp1 were pipetted directly into the lysis buffer and then treated according to the instructions of the supplier.

### PCR amplification and sequencing of genomic DNA

PCR amplification of *S. mansoni* adult worm genomic DNA or BAC DNA was performed with the Advantage 2 PCR Enzyme System (Clontech). The *Sm*PoMuc loci were amplified using *S. mansoni* adult worm genomic DNA, forward primer Intron2/3F1 and reverse primer Exon15R (see Table 1 for primer sequences and PCR cycling conditions) designed in conserved genomic regions, namely in the introns upstream of exon 2 and in exon 15, respectively (see below for primer positions). PCR products were cloned into pCR4-TOPO (TOPO TA Cloning kit for sequencing, Invitrogen). Plasmid DNA was purified using the Wizard Plus SV Miniprep DNA purification system (Promega). DNA was sequenced using a dideoxy-dye-terminator method (CEQ DTCS-Quick Start kit, Beckman Coulter) and a CEQ 8000 capillary sequencer (Beckman Coulter) with M13 forward, M13 reverse and specific primers (Table 1). Sequence analysis was performed using Sequencher software (Gene Codes Corporation).

**Table 1:** Primers used for sequencing, PCR and quantitative-PCR.

Primers		
name	sequence	cycling conditions
**For sequencing**		
Intron2/3F2	TTCTGTGTTATATACAACGTG	
Exon3F	TCCAGAACATTTGAAAACGAG	
Intron3/4R	CACATGCATAGCTAATGTGGTAATG	
Intron3/4F	AAATCGTGTGTTTATGGAATTGACG	
Exon4F	TATCTCTTGAACCATATACACGCGC	
Exon4R	GCGCGTGTATATGGTTCAAGAGATA	
Exon5F	TATTTCTTCTAGAATGTCTGAG	
Exon5R	TAGATAATGTACTGCCCACTTTGTG	
Intron5/6F	ATATGTGCGTCTGCTTTTAACTACG	
Intron6/7F	GCTGTCTCTCGCTAACAATACGACG	
Intron6/7R	ACATTTTCGTCGTATTGTTAGCGAG	
Intron7/8F1	CAGCTTCACATAAATGGAAACAC	
Intron7/8F2	AGTGGTTTACGAAAGTGAGGC	t_A_:48 - elong: 4min - 40 cycles
Intron7/8R	TAGTAACATTGGTCGTTCGTG	
Intron8/9F	AATGAAATAGTGAAAGAATGTTCG	
Intron8/9R	CTTTCACTATTTCATTCAACAACG	
Intron9/10F	CATCGCGTTATTCACTTAGCC	
Intron9/10R	TAAAGGTGGAATATGCCAAACTCAC	
Exon10F	TGAAGCTCAACTCAGTAAGCTGAAC	
Exon10R	AACTCATTATTTTGAATGTTCAGC	
Exon11R	CTTGTATCGCCTTCGATTCCAATTC	
Exon11/12F	GACAGATTCGCTTAGTGATGAAG	
Intron11/12R1	CTTCATCACTAAGCGAATCTGTC	
Intron11/12R2	GTTGCCTGAATTCACCATCTC	
Exon14F	TTCTTAGCACTACCCAAAGATGAAC	
Exon14R	TATTTGTTCATCTTTGGGTAGTGC	
Intron14/15R	GTATAATTCCTAAATATCGC	
Exon15R	TGACACAGAAAACTGTTAACGATCC	
**For PCR amplification**		
Exon1F12	GGAAGAATGAACAAGAAAATTATTCTC	t_A_: 65°C - elong: 3 min - 40 cycles
Exon15R	TGACACAGAAAACTGTTAACGATCC	
NestedExon1F	TATNTTGCGCTGATGATAAG	t_A_: 46°C - elong: 3 min - 40 cycles
NestedExon15R	ATCATAAACAAACACTGAGG	
Intron2/3F1	CACTTGTTCATAAACACGTGTCTTC	t_A_: 59,5°C - elong: 10 min - 40 cycles
Exon15R	TGACACAGAAAACTGTTAACGATCC	
Intron2/3F1	CACTTGTTCATAAACACGTGTCTTC	t_A_: 60°C - elong: 45 s - 30 cycles
Exon4R	GCGCGTGTATATGGTTCAAGAGATA	
Intron3/4F	AAATCGTGTGTTTATGGAATTGACG	t_A_: 60°C - elong: 50 s - 30 cycles
Intron3/4(gr.2)R	ATTCAAATCAGTGATTGGTGTTCAC	
Intron3/4(gr.3.4–5)R	CATGAAAATGGGTTATTTGCTAGTG	
Intron3/4(gr.3.4–5)F	CACTAGCAAATAACCCATTTTCATG	t_A_: 60°C - elong: 4 min - 30 cycles
Intron3/4F	AAATCGTGTGTTTATGGAATTGACG	
Intron9/10R	TAAAGGTGGAATATGCCAAACTCAC	
Intron5/6F	ATATGTGCGTCTGCTTTTAACTACG	t_A_: 60°C - elong: 5 min - 30 cycles
Exon11R	CTTGTATCGCCTTCGATTCCAATTC	
Intron5/6F	ATATGTGCGTCTGCTTTTAACTACG	t_A_: 60°C - elong: 50 s - 30 cycles
Intron6/7R	ACATTTTCGTCGTATTGTTAGCGAG	
Exon10F	TGAAGCTCAACTCAGTAAGCTGAAC	t_A_: 60°C - elong: 25 s - 30 cycles
Exon11R	CTTGTATCGCCTTCGATTCCAATTC	
Exon3F	TCCAGAACATTTGAAAACGAG	t_A_: 58°C - elong: 20 s - 30 cycles
Intron3/4R	CACATGCATAGCTAATGTGGTAATG	
**For intermingled repeats amplification**		
r1.F2	GCTCTCACATTTCAGATGACTAT	t_A_: 60°C - elong: 1 min - 30 cycles
r1.R2	AACTCACCTGTTGGTTCGCTC	
r2.F2	TCTCACATTTCAGGTGACCTC	
r2.R2	AACTCACCTGTGGGTTTGTCTG	
**For real time quantitative PCR**		
Exon7F2	TATACGGAACAGACATGAGC	
Exon7R	ACATTGGTCGTTCGTG	
Src.F1	TACGCTACCAACCCTGT	
Src.R1	CAAACTGCCCTTCTGT	

To identify *Sm*PoMuc genes in BACs 41B11, 62F12, 62J10, 47P6, 51E8 and 45D24, PCR amplification was performed using BAC DNA and primers (Table 1) that generate PCR fragments of different lengths for each *Sm*PoMuc group. PCR products were separated by electrophoresis in 1% agarose gels, and visualized by staining with ethidium bromide. PCR cycling conditions were one denaturation step of 1 min at 95°C followed by 30 amplification cycles: 95°C for 30 s, t_A_°C for 30 s and 68°C for a specific elongation time. t_A_°C and elongation times specific for each primer couple are given in Table 1. Cloning and sequencing was performed as described above.

To determine the presence of tandem repeats (TR) of r1, r2 and r1/r2 combinations, PCR was done with primers that bind specifically to either repeat. Amplification with r1 forward and reverse primers reveals r1 TR only, amplification with r2 specific primers shows the presence of r2 TR, and amplification with r1 forward/ r2 reverse and r2 forward/r1 reverse primers indicates the presence of r1/r2 combinations. Primers and PCR conditions used are listed in Table 1.

The copy number of *Sm*PoMuc genes was measured by quantitative PCR. Real-Time PCR analysis was performed on genomic DNAs extracted from 3 *S. mansoni* adult clones of both strains. PCR and relative quantification were performed according to previously described procedures [23],[24] with a Light Cycler (Roche Molecular Biochemicals, Germany). Specific primers for real-time quantitative PCR were designed using the Light Cycler Probe Design Software version 1.0 with an annealing temperature of 60°C. The primers used (Exon7F2 and Exon7R, Table 1) were chosen in a conserved region present in all *Sm*PoMuc genes in the intron downstream of exon 7 (see below for amplicon location). The single-copy gene used as a reference was Src kinase TK3 (GI: 37776868) amplified using primers Src.F1 and Src.R1 (Table 1). Real time quantitative PCR cycling conditions were as previously described [25].

### Nested RT-PCR on individual sporocysts of both strains

Messenger RNAs extracted from individual Sp1 were reverse transcribed by adding the enzyme mix (Superscript II, Invitrogen) directly to the paramagnetic Dynabeads. Dynabeads and associated cDNA were recovered using the magnetic system, washed twice in 10 mM Tris (pH = 7.5) and directly submitted to PCR amplification. Primers and cycling conditions used for the first round of PCR (Exon1F12 and Exon15R) and a subsequent nested PCR (NestedExon1F and NestedExon15R) are given in Table 1. All PCR were performed with the Advantage 2 PCR Enzyme System (Clontech). PCR products were separated by electrophoresis in 1% agarose gels, visualized by staining with ethidium bromide, and cloned into pCR4-TOPO for sequencing.

### Fluorescence In Situ Hybridization (FISH)

FISH was performed on *S. mansoni* sporocyst metaphase chromosome (from a Puerto Rican strain, [22]) spreads with BACs 41B11 (*Sm*PoMuc group 2, 163 A6) and 45D24 (*Sm*PoMuc group 3, 180 B12) using techniques previously described [26],[27].

### Northern Blot

Northern Blot was performed according to previously described procedure [28].

### Southern Blot

Twenty µg genomic DNA or 1 µg BAC DNA were respectively digested with 40 U or 10 U of *EcoRV*, *EcoRI* and *BclI*. DNA fragments were separated by gel electrophoresis through 0.7% agarose, and stained with ethidium bromide to confirm complete digestion. DNA was transferred to Hybond N+ membranes (Amersham Bioscience) using a vacuum blotter (model 785, Bio-Rad). Genomic repeat stretches were revealed using a 1079 bp PCR product (obtained using r2.F2 and r2.R2 primers) labeled with digoxigenin-dUTP by random priming. Hybridization and development of blots were performed with the DIG High Prime DNA Labeling and Detection Starter Kit (Roche). After stripping, the same membranes were hybridized with oligonucleotides specific for r1 and r2 repeats (CTGTTGGTTCGCTCAATGCATA, GTGACCTCGCATCAGACAAAC, respectively) 5′- labeled by DIG (Eurogentec). Positive fragments were revealed by the NBT/BCIP color reaction (Roche).

### *In-silico* analysis

Complete CDS corresponding to the three groups of *S. mansoni* mucin-like proteins (groups 1, 2, 3, [18]) were used in Blast searches against the *S. mansoni* genome (assembly version 3.1) at the Sanger Institute (http://www.sanger.ac.uk/cgi-bin/blast/submitblast/s_mansoni). Matching contigs were retrieved and genomic DNA sequences were aligned with the Sequencher software (Gene Codes Corporation). *Sm*PoMuc-containing BACs were identified by a Blast search in the BAC-ends database of the Institute for Genomic Research (TIGR) (http://www.tigr.org/tdb/e2k1/sma1/map_ends.shtml) using conserved parts of the genes.

Sequences corresponding to *Sm*PoMuc conserved genomic regions were aligned using Sequencher and manually inspected with the BioEdit Sequence Alignment Editor software (release 7.0.9.0). Parsimony trees were constructed using PAUP (Swofford, D) and robustness was checked by a bootstrap test (1000 replicates). Trees were visualized with TreeView 1.6.6. (http://darwin.zoology.gla.ac.uk/~rpage/treeviewx/index.html).

cDNA sequences were codon-aligned to the corresponding *Sm*PoMuc amino acid sequences using the PAL2NAL web server [29], and synonymous and non-synonymous substitution rates (*K_S_* and *K_N_*) were calculated essentially as described by Nei and Gojobori [30] using SNAP (http://www.hiv.lanl.gov/content/hiv-db/SNAP/WEBSNAP/SNAP.html). A test of neutrality was performed with the Neutrality Test 1.2 software (http://www.hgc.sph.uth.tmc.edu/neutrality_test/). Tajima's D test was used to detect deviation of the *K_S_ /K_N_* ratios from neutrality [31]. *Sm*PoMuc genes were annotated using SeqVISTA 1.9 software; paralogous sequence blocks were color-coded and highlighted to visualize recombination between members of the gene family.

Prediction of glycosylation sites in *Sm*PoMuc amino acid sequences was performed using the NetOGlyc 3.1 server (http://www.cbs.dtu.dk/services/NetOGlyc/) that produces neural network predictions of GalNAc O-glycosylation sites in mucin-like proteins [32].

## Results

### *Sm*PoMuc polymorphism is apparent at the protein and transcript levels

**Figure 1:**
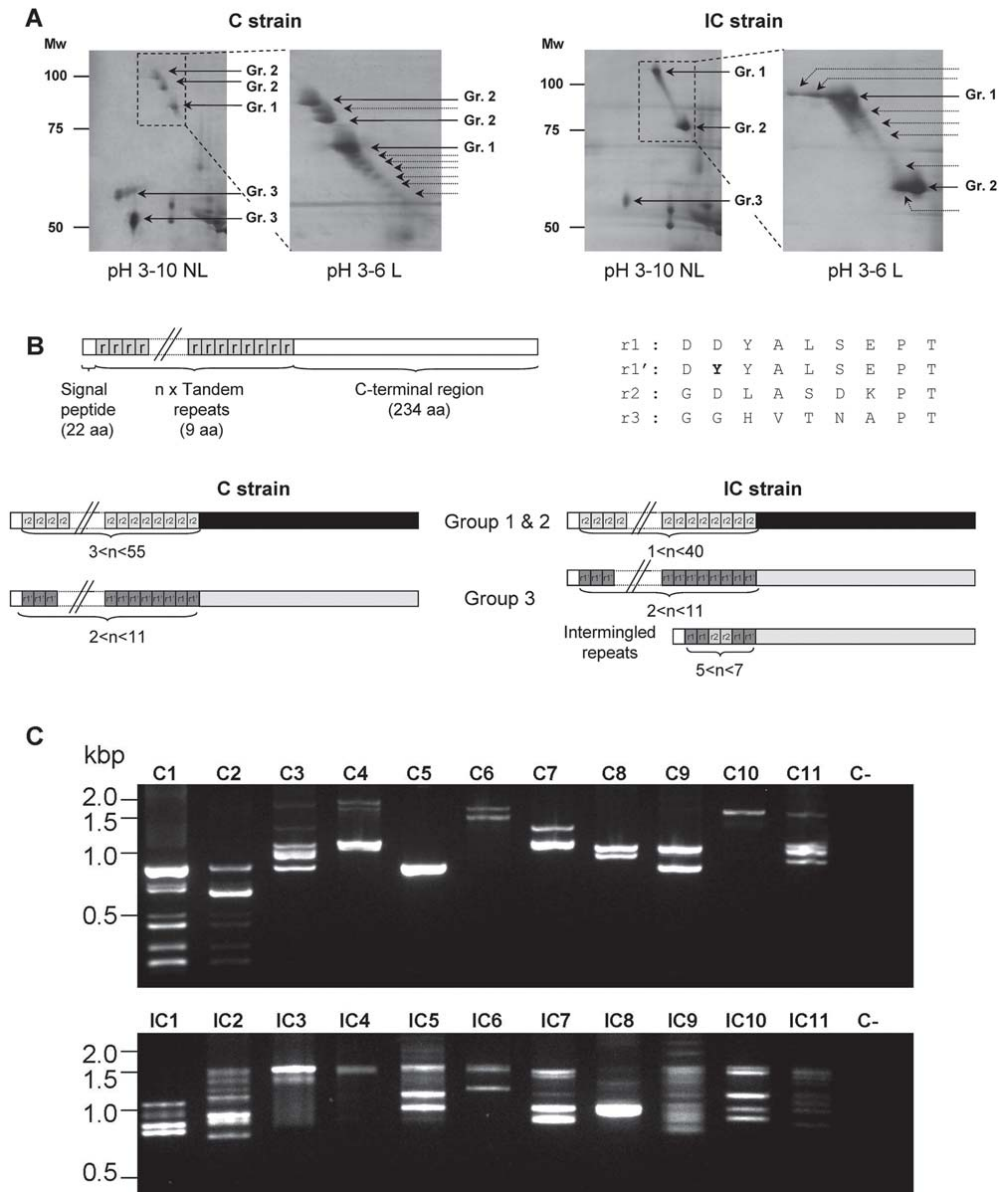
*Sm*PoMuc polymorphism at the protein and transcript levels. Positional differences between *Sm*PoMuc from compatible (C) and incompatible (IC) strains on silver stained 2D-gels shown with a pH 3–10 non-linear (NL) gradient or a pH 3–6 linear (L) gradient (A). Positions of spots corresponding to *Sm*PoMuc are indicated by arrows. Supplementary spots found in the present study using the pH 3–6 linear gradient are indicated by dotted arrows. (B) shows the precursor structure and polymorphism of *Sm*PoMuc described in a previous study [18]. Three kinds of repeats were identified in *Sm*PoMuc cDNAs (r1, r1' and r2); the fourth repeat r3 was only identified at the genomic level only in this study. (C) Agarose gel separation of RT-PCR amplicons obtained from 11 individual sporocysts (1–11) of both strains (compatible: C and incompatible: IC). Amplification was performed using consensus primers amplifying the complete coding sequence of all *Sm*PoMuc. C-: negative control of amplification.

We previously investigated differences in the proteomes of two strains of *S. mansoni* that are compatible (C) or incompatible (IC) towards a specific *B. glabrata* strain in a study that identified the *Sm*PoMuc group of proteins [17]. Evidence for *Sm*PoMuc polymorphism was provided by size and charge differences of these proteins in 2D gels (Figure 1A). To better characterize this polymorphism at the protein level, we used different pH gradients in 2-D electrophoresis. In our previous study, we had used a pH 3–10 non linear gradient to obtain a broad overview of the distribution of *Sm*PoMucs (Figure 1A) [17]. We have now realized a zoom-in gel using a narrow pH 3–6 range to expand the region containing these proteins (Figure 1A). This approach reveals several supplementary spots previously not observed and provides evidence that polymorphism at the protein level was probably underestimated in our previous study [17]. In a subsequent exhaustive analysis of *Sm*PoMuc transcripts [18] a large number of molecular variants were revealed that were classified into three groups corresponding to the different spot groups identified in the proteomic analysis. The deduced precursor sequences of the different cDNA variants are shown in Figure 1B. They are composed of a signal peptide (22 amino acids in length) followed by a variable number of tandem repeat (VNTR) domain of 9 amino acids (n = 1 to n≈55). Three different types of repeats were identified: r1, r1' and r2 that were expressed in both *S. mansoni* strains (Figure 1B). For both strains, groups 1 and 2 share common characteristics: they are always associated with r2 tandem repeats, and the number of repeats is highly variable (Figure 1B). Major differences between the strains emerge in the third group of molecular variants that are preferentially associated with r1 and r1' repeats. The same variability in repeat number as the first two groups was observed (Figure 1B) for the C strain. This is also true for the IC strain, but in contrast to C an additional sub-group exists in this strain with about half the variants containing combinations of the two types of repeats r1 (or r1') and r2 (Figure 1B). This size polymorphism of tandem repeats was also confirmed using Northern blot analysis in which a large band was obtained after hybridization with probes corresponding to *Sm*PoMuc of the first group (data not shown).

Finally, we analyzed the expression of the *Sm*PoMucs at the level of individual larvae. Consensus oligonucleotides were used to amplify the whole coding sequence of all *Sm*PoMucs by nested RT-PCR and revealed a high degree of polymorphism between individuals for the two strains (Figure 1C). This polymorphism was extensively analyzed by sequencing in the present work (see above and Table S1). Taken together, our data give evidence for a remarkable level of polymorphism of the *Sm*PoMuc molecules.

### *Sm*PoMuc genes form a multi-gene family specific for *S. mansoni*

**Figure 2:**
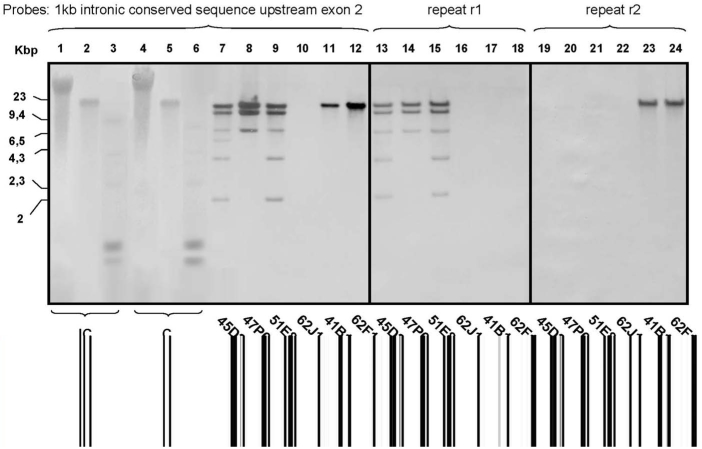
Southern blot of *S. mansoni* genomic DNA and BACs containing *Sm*PoMuc genes. Southern Blot of adult worm genomic DNA from IC (lanes 1, 2 and 3) and C (lanes 4, 5 and 6) strains and BAC clones 45D24 (lanes 7, 13, 19), 47P6 (lanes 8, 14, 20), 51E8 (lanes 9, 15, 21), 62J10 (lanes 10, 16, 22), 41B11 (lanes 11, 17, 23), 62F12 (lanes 12, 18, 24). Genomic DNA from both strains is undigested (lanes 1 and 4), digested by *EcoRV* (lanes 2 and 5) or digested by *BclI* (lanes 3 and 6). All genomic DNA lanes were hybridized with a DIG labelled probe corresponding to the 1 kbp genomic repeat shared by all *Sm*PoMuc genes. BAC DNAs were digested with *EcoRV*. Lanes 7 to 12, lanes 13 to 18 and lanes 19 to 24 correspond to the same blots hybridized successively with the 1 kbp genomic repeat, r1 and r2 probes, respectively. The membrane was stripped between two successive hybridization procedures.

**Figure 3:**
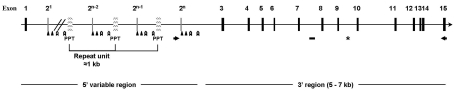
Schematic representation of a complete *Sm*PoMuc gene. The complete *Sm*PoMuc genes are composed of 15 exons. Exon 2 is included in a genomic repeat that can be repeated several times (a maximum of 20 repeats in *Sm*PoMuc 2 genes). These genomic repeats of approximately 1 kilobase are separated by imperfect polypurine tracts (PPT). Positions of genomic primers used for *Sm*PoMuc gene amplification (Intron2/3F1 – Exon15R) are indicated by arrows. PCR amplicon position used for gene copy number quantification is indicated by a bold line (–) and the position of a ribozyme between exon 9 and 10 is indicated by an asterisk. Triangles and chevrons indicate complementary sequence positions (12 and 13 nucleotides, respectively) identified in introns of the genomic repeats containing exon 2.

Based on *in-silico* investigations (Blast searches on the *S. mansoni* genome assembly v3.1, http://www.sanger.ac.uk/cgi-bin/blast/submitblast/s_mansoni/omni), we estimated the number of *Sm*PoMuc genes to be ten. Six of them correspond to full length genes (contigs Smp_contig019963, -030125, -043854, -026239, -037561, -045752) and four of them (contigs Smp_contig049466, - 010496, -045333 and - 030128) are truncated genes interrupted by a transposon insertion. To determine the number of genes in this multigene family in our strains of interest, we performed a Southern blot with DNA extracted from adult worms of the C and IC strains (200 pooled individuals for each strain) and observed one band and a smear for both strains (Figure 2, lanes 2 and 5). Since these results could be due to *Sm*PoMuc polymorphism between individuals we next analyzed *Sm*PoMuc copy number by quantitative PCR using primers designed from a conserved region of *Sm*PoMuc genes (see Figure 3 for the location of the amplicon) on DNA extracted from adult clones from both strains. Copy numbers were obtained by comparison of *Sm*PoMuc target genes to a reference gene that was confirmed to be single-copy per haploid genome (*Schistosoma mansoni* Src kinase TK3, [33]). Our findings indicate that the number of *Sm*PoMuc genes varies from 6 to 9 depending on individuals tested for both strains. These results are in agreement with the gene number (6) identified in the *S. mansoni* genome assembly database since the primers used for copy number quantification do not amplify truncated genes.

We then investigated the structure of *Sm*PoMuc genes based on sequences available in the genome assembly database. *Sm*PoMuc genes are composed of 15 exons of an average size of 60 bp. Intron average size is approximately 550 bp and varies between *Sm*PoMuc genes because of insertion/deletion events.

The striking feature of the 5′ variable region spanning exons 1–2 is that exon 2 and its flanking introns occur as tandem repeats. These genomic repeats of approximately 1 kb are separated by microsatellites and we discuss below the detailed description and high level of similarity of these genomic repeats between all members of *Sm*PoMuc multigene family. This conservation prevented the assembly of this region of the *Sm*PoMuc genes and explains their frequent incomplete assembly into contigs in the databases.

**Figure 4:**
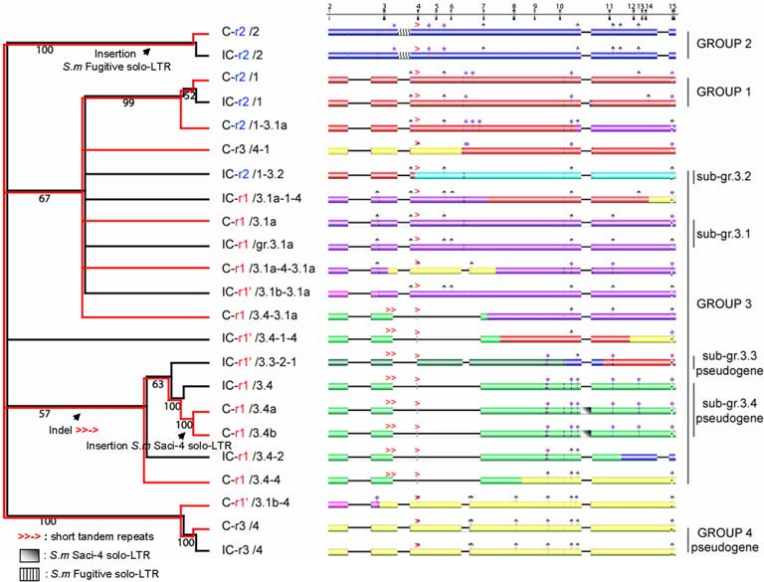
The *Sm*PoMuc multigene family is organized in four paralogous groups that frequently recombine. *Sm*PoMuc genomic DNA sequences corresponding to the 3′ portion of *Sm*PoMuc genes/alleles (exon 2/exon 15) were obtained by long range PCR and aligned to construct a cladogram with PAUP. Tree branches corresponding to C and IC strains are in red and black, respectively. *Sm*PoMuc genes are identified as follows: first the strain (C or IC), then the last exon 2 (r1, r1' or r2) and finally the group (1, 2, or 4) or sub-group (3.1a, 3.1b, 3.2, 3.4, 3.5). This analysis reveals four paralogous sequence groups (gr.1–gr.4). In the right-hand part of the figure, a schematic representation of aligned *Sm*PoMuc genomic sequences is given. We annotated the sequences by a color code that uses a different color for sequence fragments of less than 95% identity: gr.1 (red), gr.2 (blue), sub-gr.3.1a (purple), sub-gr.3.1b (pink), sub-gr.3.2 (sky-blue), sub-gr.3.3 (dark-green), sub-gr.3.4 (green) and gr.4 (yellow). Traces of retrotransposon insertion events (solo-LTR) are present in sub-gr.3.4 and gr.2. Large gaps necessary to obtain alignments are represented by dark lines. Short gap (<28 nucleotides) positions are indicated by rhombi. Short tandem repeats are indicated by (>). Frequent recombination events between *Sm*PoMuc family members are apparent.

To investigate the different genes and/or alleles in our strains of interest and the relationships between individual members of this gene family, we performed an analysis of PCR-amplified, subcloned and aligned *Sm*PoMuc genomic DNA sequences and constructed a cladogram with PAUP (Figure 4). The genomic sequences used for this analysis correspond to the 3′ part of the genes and were obtained using universal primers amplifying *Sm*PoMuc genes between the last exon 2 and exon 15 (see Figure 3 for primer positions) in both strains. Respectively 12 and 11 different sequences were obtained for C and IC strains, (GenBank accession numbers EU676572 to EU676594). The unrooted tree option was chosen since no genomic sequences with reasonable similarities to *Sm*PoMuc are available in the databases. Our analysis shows clearly that the *Sm*PoMuc gene family can be divided into four paralogous sequence groups (gr.1–gr.4) that are closely related for both strains (Figure 4). Essentially, these 4 groups were formed by insertion/deletion (indel) events in the non-coding regions and subsequent gene duplications. Group 3 can be divided into 5 sub-groups (sub-gr.3.1a, -3.1b, -3.2, -3.3, -3.4). Sub-gr.3.1a and -3.1b differ only by sequences located in introns. Sub-gr.3.1 and -3.2 share the same structure as the other groups but their sequences are different. Sub-gr.3.3 and -3.4 have undergone large deletion events in intronic and intronic/exonic regions, respectively. Traceable indel events like solo-LTRs, whose formation involves recombination between the LTRs of retrotransposons [34], are observed, for example the creation of C-r1/3.4a and C-r1/3.4b in sub-group 3.4 by insertion of a Saci-4 solo-LTR between exons 10 and 11 (Figure 4). We also identified short tandem repeats flanking some deleted sequences (e.g. between exons 3 and 4 in several *Sm*PoMuc genes). Traceable indel events are annotated in Figure 4.

A single group of truncated genes was identified. In these genes of the 3.4 sub-group, exons 4, 5 and 6 were deleted. Other truncated genes or pseudogenes were not included in the analysis since they were not amplified with the primers used. In the *S. mansoni* genome assembly (version 3.1, Sanger Institute http://www.sanger.ac.uk/cgi-bin/blast/submitblast/s_mansoni), four truncated genes/pseudogenes could be identified by BLAST searches (contigs Smp_contig049466, - 010496, -045333 and - 030128). However, the current assembly status of the *S. mansoni* genome does not allow for reliable statements concerning these loci because the succession of exons is interrupted by transposon insertion events and these transposons are very conserved and repeated frequently in the *S. mansoni* genome.

Three groups of paralogous *Sm*PoMuc genes (gr.1, gr2 and sub-gr.3.1) correspond to the three groups of cDNA variants we found in a previous study [18]. These three groups include genes that are expressed in both strains (Figure 1B). The genes that belong to the fourth group and sub-gr.3.3 and 3.4 are probably pseudogenes because their transcripts were not detected either in our previous work [17],[18] or in the present study. In addition, the gr.4 *Sm*PoMuc genes are associated with a different exon 2 (repeat r3, Figure 1B) that was itself never detected in a transcript. The sequence of this particular exon 2 is given in Figure 1B to allow comparison with other cDNA repeats in *Sm*PoMuc transcripts.

**Figure 5:**
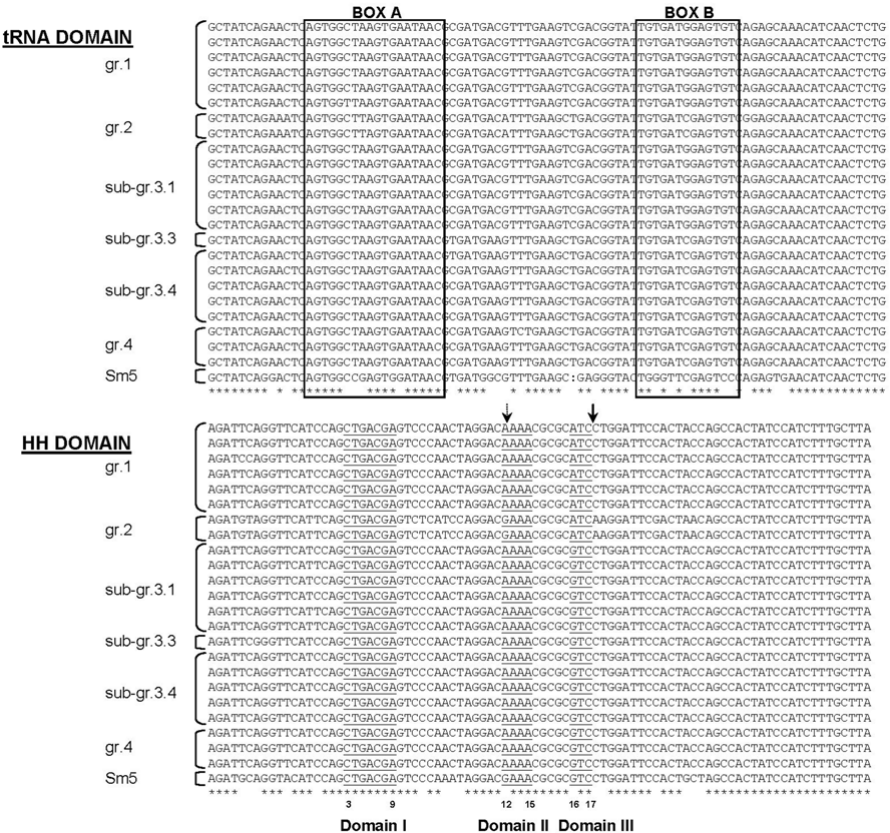
*Sm*PoMucs contain a putative full-length hammerhead ribozyme between exon 9 and 10. Alignment of putative ribozymes found in all *Sm*PoMuc genes with a functional hammerhead ribozyme of *S. mansoni* (Sm5, AF036742). Asterisks indicate conserved positions in the alignment. Boxes A and B delimit sequences necessary for transcription by RNA polymerase III. The catalytic core nucleotides composed of domains I, II and III are underlined. The conserved nucleotides are numbered using the standard convention [74]. The nucleotide position corresponding to G12 essential for ribozyme activity [39] is indicated by a dotted arrow. The scissile bond is indicated by an arrow.

Finally, another interesting characteristic of these genes is that they all contain an intron-coded hammerhead ribozyme between exon 9 and 10. *S. mansoni* hammerhead ribozymes were extensively studied and shown to catalyze cleavage [35] and ligation [36] of transcripts *in vitro*. We have aligned the different ribozymes obtained for the 23 genes sequenced in both strains with the sequence of the *S. mansoni* hammerhead ribozyme that is the most extensively studied (GenBank accession number: AF036742; [36]–[38]). The alignment (Figure 5) shows that all the genes possess the promoter elements (boxes A and B) that are essential for transcription by RNA polymerase III [37]. The aligned sequences correspond to natural ribozymes that display the canonical structure of schistosome hammerhead ribozymes consisting of three helices and a catalytic core. However, only the putative ribozymes of the *Sm*PoMuc 2 group possess the G12 of the catalytic core that was shown to be essential for activity [39].

### *Sm*PoMuc genes recombine frequently and evolve under selective pressure

**Figure 6:**
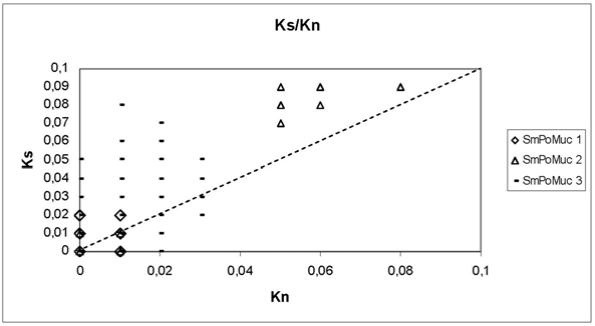
*K_S_/K_N_* comparison of *Sm*PoMuc coding sequences. The analysis was performed using SNAP (see Material and Methods) on 15, 71 and 56 sequences from groups 1, 2 and 3 respectively. The closed rhombi, open triangles and dashed lines are used for a pair of *Sm*PoMuc sequences from groups 1, 2 and 3, respectively. The bisecting dotted line corresponds to *K_S_/K_N_* = 1.

To analyze whether recombination events occur between *Sm*PoMuc genes, we annotated the available sequences by a color code that uses a different color for sequence fragments of less than 95% identity (Figure 4). By visual inspection we identified at least 14 recombination events between the 23 genes amplified by PCR in the 3′ constant region amplified by PCR. Recombination break points are evenly distributed along the sequence. We noted that these recombination events can generate mosaic genes, the sequences of which could originate from the different members of the multi-gene family. For example, exons from the gr.4 *Sm*PoMuc pseudogenes can be found in several of these mosaic genes belonging to gr.3 (Figure 4). We then investigated whether all *Sm*PoMuc genes are under selective pressure and calculated ratios of synonymous to non-synonymous substitutions (*K_S_/K_N_*) in 15, 71 and 56 subcloned RT-PCR products for the genes of groups 1, 2 and 3 respectively. This analysis was performed on cDNA sequences (Genbank accession numbers EU676503 to EU676571 and EU042599 to EU042636) in the 3′-terminal conserved part of *Sm*PoMucs. As shown in Figure 6, the *K_S_/K_N_* ratio is >1 in a large majority of the transcripts (93.1%, 86.8% and 90.8% for groups 1, 2 and 3 respectively), indicating that all genes are under selection. Likewise, Tajima's test for neutrality delivers significant negative D values indicating that a purifying selection acts within the three groups of *Sm*PoMuc (D1 = −2.305, D2 = −2.58, D3 = −2.77 and *p* value<0.05). Therefore, all *Sm*PoMuc genes have evolved under selective pressure.

### *Sm*PoMuc tandem repeats have unique features

As described above, the part of the genes encoding the repeat units of the protein are composed of repetitive units of approximately 1 kb including an exon of 27 bp (corresponding to exon 2) and separated by microsatellites (see Figure 3). These microsatellites are considered as imperfect because they are composed essentially of dinucleotide repeats (GA) whose succession is often interrupted. To analyze the diversity of these genomic repeats, we analyzed all trace records containing exon 2. Their size ranges from 1022 to 1037 bp differing by the size of the microsatellite. The intronic part of the repeat units is highly conserved, displaying more than 94% identity and prevents assembly of the trace records corresponding to this part of the *Sm*PoMuc genes. PCR amplification, cloning and random sequencing (31 clones for each strain, GenBank accession numbers: EU676595 to EU676656) of the DNA of our strains of interest reveals the same size window (about 1070 pb) and the same level of conservation (>93%).

Since assembly of trace records was impossible in this repetitive 5′ region of *Sm*PoMuc genes, we were unable to determine the number, size and nature of repeats associated with the different *Sm*PoMuc genes by *in silico* analysis. To circumvent this problem, we analyzed *Sm*PoMuc-containing BACs from the Sm1 library [22]. The BACs of interest were selected by Blast searches against the TIGR BAC ends database. Positive clones were recovered and digested with *EcoR*V that does not cut within the repeat units. Fragments were size separated by gel electrophoresis and hybridized with (i) a probe directed to a conserved intronic sequence upstream of exon 2, or (ii) probes that distinguish between r1 and r2 repeats. Results are shown in Figure 2. Each band corresponds to an intact stretch of repeat units and two types of restriction (data not shown) and hybridization patterns can be clearly distinguished. BACs 45D24, 47P6 and 51E8 span a genomic region in which at least 6 polymorphic *Sm*PoMuc loci are situated (Figure 2, lanes 7-8-9). This result was confirmed by PCR amplification of BACs and sequencing of the PCR products: the number of sequences obtained corresponds to the number of bands observed for each BAC clone on the Southern blot (Table 2). This cluster contains at least 6 tandemly repeated *Sm*PoMuc group 3 genes. In addition, all loci of this cluster exclusively contain r1 type repeats (Figure 2, lanes 13-14-15). The second group of BACs contains one or two genes of *Sm*PoMuc group 2 (confirmed by PCR and sequencing (Table 2)). BACs 41B11 and 62F12 contain a single *Sm*PoMuc locus or multiple monomorphic loci with r2 repeats only (Figure 2, lanes 23–24). BAC 62J10 contains none of the repeat sequences (Figure 2, lanes 16–22). PCR confirmed the presence of a truncated gene without genomic repetitive units on this BAC. This truncated gene is also present in the two other BACs spanning this genomic region. It corresponds to a duplicated truncated form of the gene *SmPoMuc 2* in the contig Smp_contig010496 of genome assembly version 3.1.

**Table 2:** *Sm*PoMuc genes contained in the different BAC clones.

45D24		47P6		51E8		62J10		41B11		62F12	
TR	*Sm*PoMuc	TR	*Sm*PoMuc	TR	*Sm*PoMuc	TR	*Sm*PoMuc	TR	*Sm*PoMuc	TR	*Sm*PoMuc
7 or 15 r1	**gr. 3.1b**	7 or 15 r1	**gr. 3.1b**	7 or 15 r1	**gr. 3.1b**	none	**Trunc gr.2**	≈20r2	**gr. 2**	≈20r2	**gr. 2**
7 or 15 r1	**gr. 3.1a**	7 or 15 r1	**gr. 3.1a**	7 or 15 r1	**gr. 3.1a**			none	**Trunc gr.2**	none	**Trunc gr.2**
1 or 2 r1	**gr. 3.3**	1 r1	**gr.3.4**	1 or 2 r1'	**gr. 3.3**						
1 r1'	**gr. 3.1b**			1 or 2 r1'	**gr. 3.3**						
1 or 2 r1'	**gr. 3.3**			1 r1	**gr.3.4**						
1 r1	**gr.3.4**										

Since DNA from C and IC strains were digested with a restriction enzyme that does not cut within the repeat units (*EcoR*V), hybridization with a probe directed to the conserved intronic sequence upstream of exon 2 reveals the genomic repeat stretches of all genes present in the DNA. This Southern blot shows that the labeled fragment size never exceeds 20 kb (Figure 2, lanes 2 and 5) corresponding to a maximum of 20 repeat units per gene for both strains. These results are in agreement with the results obtained with the BACs in which the longest fragment corresponding to *Sm*PoMuc repeat stretches again never exceeded 20 kb. In addition, most loci present in BACs 45D24, 47P6 and 51E8 contain less than 10 repeat units (Figure 2, lane 7-8-9). This contrasts strikingly with the transcripts in which up to 100 repeat units can be present (see below).

**Figure 7:**
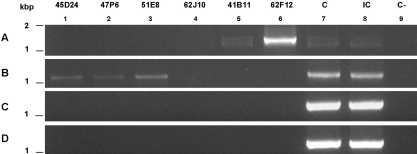
Intermingled repeats (r1/r2) are present in C and IC genomic DNA but not in BACs. PCR experiments were performed on BACs 45D24 – 47P6 – 51E8 – 62J10 – 41B11 – 62F12 (lanes 1 to 6, respectively) and on DNA from C and IC strains (lanes 7 and 8, respectively); lane 9 corresponds to the PCR negative control. Amplicons were separated on TAE 1% agarose gels and revealed by ethidium bromide staining. The primers used reveal two r2 exons (A), two r1 exons (B), r2r1 exons (C) or r1r2 exons (D) in two successive genomic repeats.

Since we have found combinations of r1 and r2 repeats in transcripts, we tested, using PCR and primers that are specific for these repeats, whether they could be amplified from two neighboring genomic repetitive units. In the case of the BAC clones, this analysis confirmed the results of the Southern Blot: TR stretches are composed of either r1 (Figure 7B, lanes 1-2-3) or r2 (Figure 7A, lanes 5 and 6) but never of both repeat units intermingled (Figure 7C and D, lanes 1-2-3-4-5-6). However, when the same PCR experiments were conducted on genomic DNA from our two strains of interest, intermingled repeats were detected in some *Sm*PoMuc genes of both strains Figure 7C and D, lanes 7 and 8). This result is in agreement with cDNA sequencing showing that intermingled repeats are regularly detected in the IC strain but also once in the C strain (see Table S1, individual C-8). These latter results also show that genes containing intermingled repeats are present in both strains but are seldom expressed in C strain individuals. In addition, we show that intermingled repeats are not detected in *Sm*PoMuc-containing BACs from the Sm1 library prepared with DNA of a Puerto Rican strain of *S. mansoni*. Moreover, intermingled repeats are detected neither in contigs from the *S. mansoni* genome assembly nor in ESTs obtained from BH and PR isolates of *S. mansoni* [40]. Therefore, intermingled repeats r1 and r2 seem to be a unique feature of our model strains.

Concerning the different genes associated with the two BAC groups, the combination of PCR analysis and sequencing (Table 2), band lengths obtained by Southern blots (combinations of restriction digests with *EcoR*V, Figure 2 and *EcoR*I, data not shown) and *in silico* analysis of genome assemblies permit us to assign the different bands to their corresponding genes: the first group (BACs 45D24, 47P6 and 51E8) spans a genomic area with at least 6 genes in tandem. All these genes belong to group 3: one gene of the 3.1a sub-group containing 7 genomic tandem repeats; two genes of the 3.1b sub-group, one containing 15 repeats and the other 1 repeat; two genes of the 3.3 sub-group containing one or two repeats; and one gene of the 3.4 sub-group containing one repeat only. The second group of BACs (62J10, 41B11 and 62F12) spans a genomic area with at least two group 2 genes: one of them is truncated and does not possess the genomic tandem repeat region; the other contains approximately 20 repeats.

### *Sm*PoMuc genes are organized in four locations on chromosome 3 and 4

**Figure 8:**
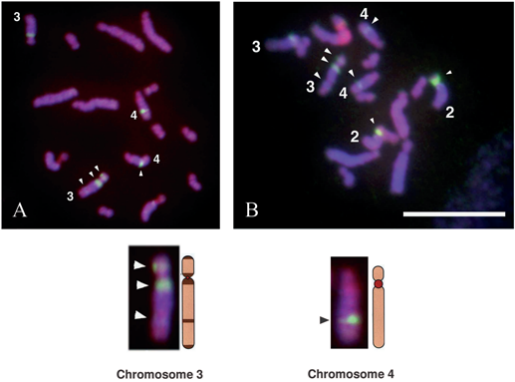
FISH mapping of *Sm*PoMuc BACs clones. Metaphase chromosome spreads showing positive signals (arrowheads) hybridized with biotinylated *Sm*PoMuc BAC clone DNAs. (a) BAC clone 41B11 gave strong signals in the regions near the centromere of chromosome 3 and on the long arm of chromosome 4; two weaker signals were also detected on the short and on the long arm of chromosome 3. (b) BAC clone 45D24 hybridized to the same regions on chromosome 3 and 4, and yielded a strong supplementary signal at the large heterochromatic pericentromeric region of chromosome 2. This last signal is probably due to repetitive sequences in this BAC and not to the presence of *Sm*PoMuc genes.

FISH on metaphase chromosomes of *S. mansoni* with BACs that are representative of each group identified by Southern blot (41B11 and 45D24) revealed the presence of four genomic *Sm*PoMuc locations. Hybridization with BAC 41B11 gave strong signals near the centromere regions of chromosomes 2 and 3 and on the long arm of chromosome 4. Two weaker signals were also detected on the short and on the long arm of chromosome 3 (Figure 8A). BAC 45D24 hybridized to the same regions on chromosomes 3 and 4, but gave no signal at the large heterochromatic pericentromeric and nucleolus organizer regions of chromosome 2 (Figure 8B). Consequently, the signal on chromosome 2 is specifically obtained only for 41B11 and is probably due to repetitive sequences in this BAC and not to the presence of *Sm*PoMuc genes. FISH thus indicates the existence of at least four distinct locations of *Sm*PoMuc genes in the genome of *S. mansoni*. Differences in signal intensity suggest that the loci near the centromere on chromosome 3 and on the long arm of chromosome 4 could contain more *Sm*PoMuc genes than the others, which is in good agreement with Southern blotting results: BACs 45D24, 47P6 and 51E8, derive from a genomic area that contains at least 6 tandemly oriented group 3 *Sm*PoMuc genes (Figure 4). The other group of BACs (41B11, 62J10, and 62F12) covers a genomic area containing at least two group 2 genes (Figure 4). *Sm*PoMuc genes of groups 1 and 4 were not identified in any of these BACs. Nevertheless, these genes were identified in the *S. mansoni* genome assembly (contigs Smp_contig019963 and -026239) as well as in our two strains of interest. These results suggest that they may be present in the two other locations identified by FISH.

### *Sm*PoMuc transcription patterns are highly polymorph, strain- and individual-specific: involvement of expression polymorphism, alternative splicing, aberrant splicing and exon repetition

In our previous studies of *Sm*PoMucs, polymorphism was investigated at the transcript level. PCR amplification with consensus primers of cDNA pools (obtained after reverse transcription of RNA extracted from one thousand sporocysts) from both *S. mansoni* strains showed distinctive banding patterns after analysis in agarose gels [18]. To address the question, whether each individual sporocyst transcribes all strain-specific *Sm*PoMuc loci, or whether expression patterns are individual-specific, RNA was extracted from 11 single sporocysts (from each strain) and nested RT-PCR was performed on each individual. Banding patterns in agarose gels indicate clearly that each individual sporocyst expresses a characteristic subset of *Sm*PoMuc genes (Figure 1C). We never detected the same pattern in different individuals, suggesting a high level of transcript polymorphism within the tested *S. mansoni* populations. PCR products of these individuals were subcloned and 20 clones of each individual were sequenced for both strains. The results are summarized in Table S1.

Our results revealed first an extensive expression polymorphism between individuals. Some of them express only genes belonging to one group (see Table S1, individuals C-2, -3, -5, -7, -8, -9, -10 and IC-1, -3, -4, -6, -11), others express genes belonging to two different groups. In this latter case, they express either genes from group 2 and sub-group 3.1 (C-1, -4 and IC-2, -5, -7, -8, -9, -10), or genes from groups 1 and 2 (C-6), or genes from group 1 and sub-group 3.1 (C-11). Of the 11 individuals, none from the IC strain express group 1 genes. This observation is not in agreement with data obtained using cDNA pools from one thousand individuals that showed that genes from this group are indeed expressed in the IC strain ([18] and Figure 1B). The result obtained on individual parasites is probably due to sampling.

**Figure 9:**
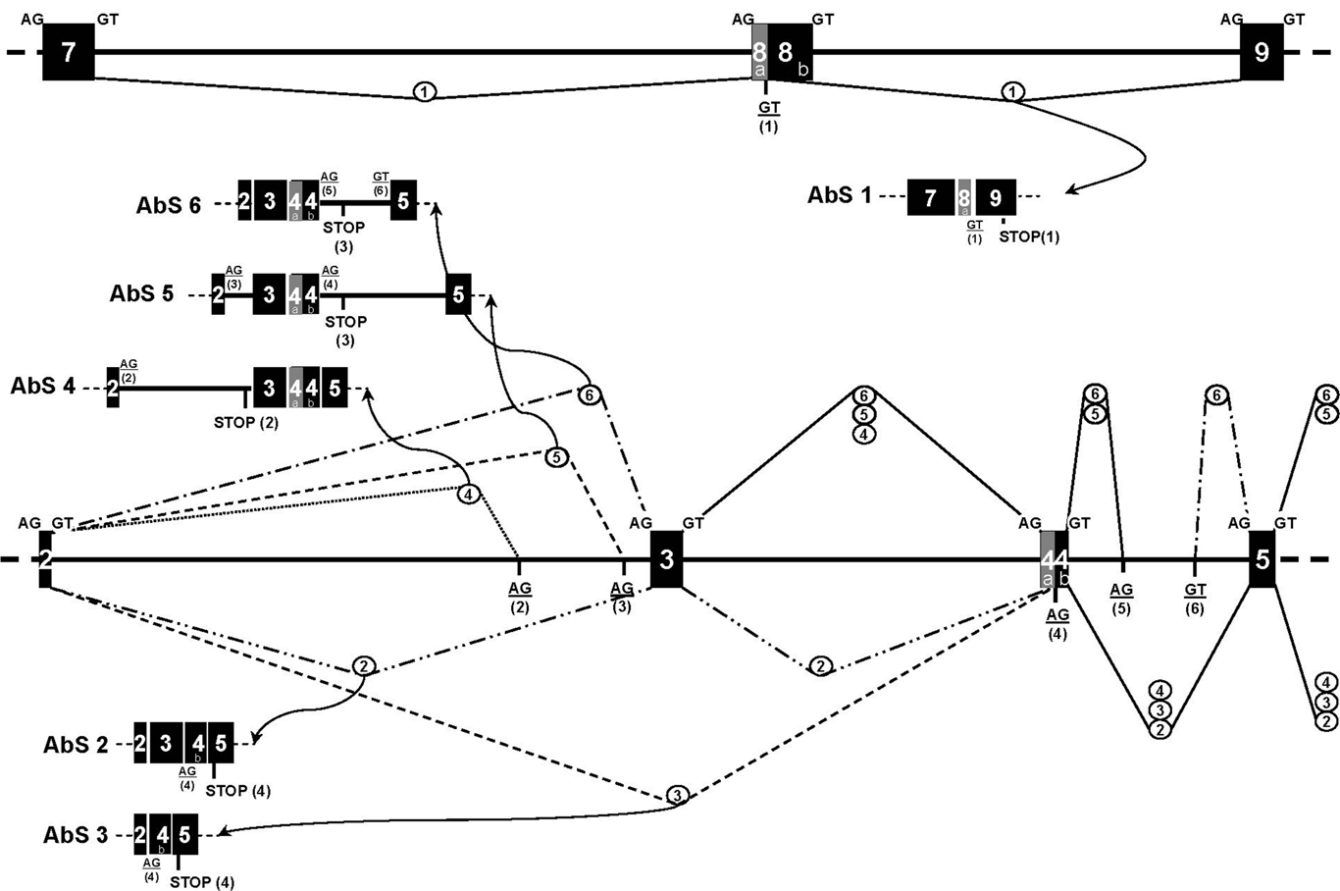
Aberrant splicing events during *Sm*PoMuc gene expression. The six aberrant splicing variants (AbS) obtained at the cDNA level are shown and numbered (AbS 1 to 6). The *Sm*PoMuc genomic sequence areas subject to aberrant splicing are shown: introns are represented as thick lines and rectangles represent exons numbered as described in Figure 3. Normal splice donor and acceptor sites are in uppercase above the schematic gene representation. Aberrant splice sites are in uppercase, underlined, numbered (in brackets) below the schematic representation of *Sm*PoMuc genes or in AbS 1–6. The different splicing events leading to them are indicated by dotted or full lines linking the different splice sites. These events are identified by circled numbers corresponding to the AbS they produce. Resulting aberrant splicing leads to exclusion (1-2-3) or inclusion (4-5-6) of DNA, leading to frame-shifts that create non-sense codons in all cases. The different aberrant splicing events observed correspond to cDNA variants given in Table S1: AbS 1 (individuals C3/2, IC/2-4-6-7-8-9-11/2); AbS 2 (individual IC11/2/7r2); AbS 3 (individual IC2/2/4r2); AbS 4 (individual IC5/3.1/10r1); AbS 5 (individual IC5/3.1/11r1) and AbS 6 (individual IC5/3.1/12r1).

Second, this analysis of cDNA reveals non-classical splicing events that occur frequently in the 3′constant region of *Sm*PoMuc transcripts. The frequency of these events is much higher in individuals from the IC strain (10/11) compared to C strain sporocysts (4/11). Some of these events correspond to alternative splicing that leads to exon deletion(s) without modifications of the Open Reading Frame (ORF). These events are observed in transcripts corresponding to group 2 and sub-group 3.1 genes. Deletions involve either several exons (*Sm*PoMuc2 and 3.1 variants of C-1 and C-2 and *Sm*PoMuc2 variant of IC-5, Table S1), or only one exon, such as exon 3, (*Sm*PoMuc2 variant of IC-2, Table S1), 10 (*Sm*PoMuc3.1 variants of IC-1, Table S1) or 8 (*Sm*PoMuc2 variant of C-4, IC-7, -9 and -10, Table S1). Other variants display aberrant splicing that changes the ORF and results in premature stop codons. This phenomenon is essentially observed in transcripts corresponding to genes of the second group and is much more frequent in variants cloned from IC strain individuals. Type 1 aberrant splicing described in Figure 9 is observed for *Sm*PoMuc2 transcripts of 8 out of 11 IC strain individuals, but only for 1 out of 11 individuals of the C strain. In addition, other types of aberrant splicing variants (types 2 to 6) were observed at low frequency. Data are summarized in Figure 9.

Finally, our individual-level analysis of expression patterns of *Sm*PoMuc reveals several novel points concerning the 5′ VNTR region, the most striking concerning transcript length. The number of tandem repeats (TR) in transcripts of the different *Sm*PoMuc genes varies from 1 to 100. In contrast, our data obtained by Southern blotting on genomic and BAC DNA (Figure 2, lanes 2–5, lanes 7-8-9-11-12) indicate a maximum number of only 20 exons 2 in the *Sm*PoMuc genes. The presence of more than 20-fold repetitions of exon 2 in over 50% of the *Sm*PoMuc2 transcript variants and the occurrence of variants that differ only by the number of repeats in some individuals (see Table S1, individual C-3 for example) shows that a trans-splicing mechanism occurs during *Sm*PoMuc transcription.

### Polymorphic *Sm*PoMuc-specific repeat patterns and splicing variations provide a basis for polymorphic glycosylation

The p*I* of *Sm*PoMuc proteins predicted from the cDNA sequences varies between 4.3 and 5 and these values are in good agreement with those determined by IEF. In contrast, the calculated molecular weights of 30 to 80 kDa, depending on the variation in length of the repeat domain, do not correspond to the measured values of between 55 and 130 kDa (Figure 1A). In a previous study we showed that *Sm*PoMucs are glycosylated and it is therefore probable that the observed molecular weight shift is due to a high degree of glycosylation in the TR [18]. Three different types of repeats were identified: r1, r1' and r2 (Figure 1B). Two of them (r1 and r1') are very similar, differing only by 1 amino acid residue and all contain S, T and P residues. Such repetitive structures with similar amino acid compositions were described in different O-glycosylated mucins, and we predicted O-glycosylation of T or S residues in these repeats (a typical feature of mucins) using the NetOGlyc 3.1 server (http://www.cbs.dtu.dk/services/NetOGlyc/).

We applied this prediction tool to the different variants obtained in the present study using the amino acid sequences deduced from these different variants. We considered that glycosylation occurs when the prediction score is superior to 0.5 as suggested by Julenius *et al.* (personal communication and [32]). The results are summarized in Table S2. They show that the predicted glycosylation status is dependent on the number and type of repeats and the type of arrangement (alternation of repeat types). These results suggest that TR polymorphism and length could be linked to glycosylation polymorphism associated with the different expressed *Sm*PoMuc variants. In addition, alternative splicing and aberrant splicing events that delete portions of the C-terminal part of the protein have a marked influence on the glycosylation prediction, for instance in the case of variants IC2/2/25r2.1 and.2 expressed by the IC-2 individual (Table S2). Aberrant splicing in the latter produces a stop codon that shortens the deduced peptide sequence. The consequence for the glycosylation prediction status is radical since the first variant contains no predicted glycosylation while glycosylation is predicted at 22 sites in the second. Because variants from IC strain individuals are more frequently subject to this type of splicing event, the number of predicted glycosylated variants is much higher (59 glycosylated variants out of 84, each containing an average glycosylation number of 8.4±7.6) than that of C individuals (10/41, each containing an average glycosylation number of 8.44±8.7).

**Figure 10:**
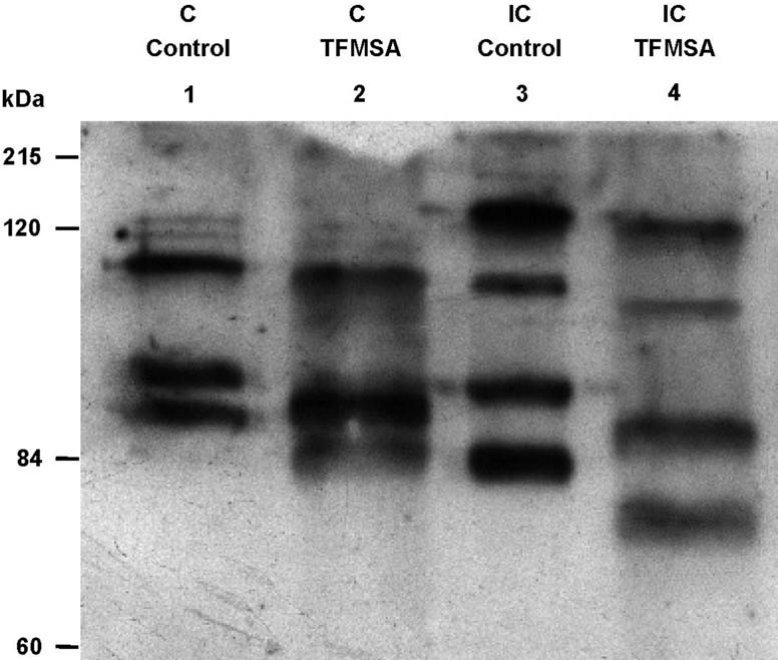
Western blot of *Sm*PoMuc proteins from C and IC strain before and after deglycosylation. *S. mansoni* sporocyst extracts from C (lanes 1-2) and IC (lanes 3-4) strains were treated with TFMSA (lanes 2–4) or not (lanes 1–3) and submitted to a western blotting using anti-*Sm*PoMuc antibodies. The shift in molecular weight observed in lanes 2 and 4 is related to the loss of carbohydrate chains associated with *Sm*PoMuc proteins.

To determine whether these glycosylation predictions had biological significance we chemically deglycosylated sporocyst extracts using trifluoromethanesulfonic acid (TFMSA) before western blotting with antibodies directed against *Sm*PoMuc proteins. Molecular weight shifts related to the loss of carbohydrate chains after deglycosylation were more marked for the IC strain (Figure 10, lane 4) than for the C strain (Figure 10, lane 2), compared to the respective controls. This suggested that the glycosylation prediction obtained following NetOGlyc 3.1 analysis was correct and that *Sm*PoMucs from the incompatible IC strain are indeed more highly glycosylated than those from the compatible C strain. The total removal of carbohydrate moieties from *Sm*PoMuc glycoproteins by TFMSA treatment was confirmed by the observation that no bands were detected in treated samples compared to controls on Alcian blue stained gels or following lectin blotting (not shown).

## Discussion

*Sm*PoMucs are mucin-like molecules that we recently discovered by a proteomics approach aiming at identifying molecular determinants of compatibility polymorphism in the interaction between *S. mansoni* and its intermediate host *B. glabrata* [17]. The comparison of the proteomes of sporocysts (intramolluskan stage) of two *S. mansoni* strains, one compatible with a specific strain of *B. glabrata*, the other incompatible with the same mollusk strain, showed that the principal difference lies in this protein family with the characteristics of mucins. We showed that these proteins are glycosylated, expressed in the apical gland of *S. mansoni* miracidia and sporocysts and are present in their Excretion-Secretion products [18]. These molecules are highly polymorphic and we therefore called them *Sm*PoMucs for *S. mansoni* polymorphic mucins. In the present study, we have extended the analysis of their polymorphism and show that each individual larva expresses a unique combination of *Sm*PoMucs derived from a limited set of genes. This extraordinary level of polymorphism may be linked to (i) gene structure, organization and evolution, and/or (ii) different regulation processes occurring during gene expression. In this study we have elucidated the complex cascade of mechanisms that confer polymorphism to *Sm*PoMuc.

*Sm*PoMucs are coded by a multi-gene family that contains at least 6 to 9 members in our strains of interest. PCR with consensus primers amplifying the 3′ part of the genes revealed a dozen sequences corresponding to different genes/alleles that can be divided into four paralogous sequence groups (gr.1–gr.4). The first three groups correspond to expressed genes for which we detected transcripts. In addition, their corresponding proteins were identified in a previous study [18]. Transcripts and proteins from the fourth group and certain subgroups of the third group were never found, suggesting that these genes are probably pseudogenes. We also identified additional pseudogenes corresponding to truncated forms of *Sm*PoMuc in the *S. mansoni* genome assembly database. These latter observations suggest that a large proportion of the genes belonging to this multigene family are non-functional, exactly as would be expected for multigene families that conform to the birth-and-death model of evolution [41].

We also found evidence for frequent recombination events between pseudogenes, especially from gr.4 and other members of the multigene family (see Figure 4). This suggests that these pseudogenes can provide an additional pool of genetic variability for the generation of new variants through recombination, gene conversion or exon shuffling. This type of variation-generating mechanism was observed for the *Trypanosoma brucei* variant membrane surface glycoprotein [42], *Anaplasma marginale* membrane surface proteins [43] and MHC [44]. The numerous insertion/deletion events identified in *Sm*PoMucs, the solo-LTR identified in some genes (gr.2 and sub-gr.3.4, Figure 4), the truncated genes interrupted by retrotransposition events (contigs identified in the genome assembly) and short tandem repeats flanking some deleted sequences (e.g. between exon 3 and 4 in several *Sm*PoMuc genes, Figure 4) illustrate these frequent reshaping events occurring in *Sm*PoMuc genes. These structural characteristics suggest that retrotransposons could play a central role as mediators of recombination between *Sm*PoMuc genes.

FISH experiments revealed that *Sm*PoMuc genes are distributed at four locations in the genome of *S. mansoni*. Two of these locations were analysed in detail using the corresponding BACs. Our analysis shows that *Sm*PoMuc gr.2 and gr.3 genes are organized in clusters in two distinct genomic locations. The *Sm*PoMuc2 cluster is composed of at least two genes, one complete containing all exons (1 to 15) and approximately 20 repeats of exon 2, and a truncated gene with no tandem repeats. The *Sm*PoMuc3 cluster is composed of at least six tandemly organized genes containing 1 to 15 exon 2 repeats. It is noteworthy that some individuals might possess fewer genes in these latter clusters as we have evidenced a gene copy number variation between individuals (6 to 9 copies of *Sm*PoMuc genes per individual). Furthermore, *Sm*PoMuc 2 and 3 clusters are associated with a specific exon 2, the first containing only r2 exons and the second only r1 exons, a result confirmed by PCR on BAC clones. Nevertheless, intermingled r1 and r2 repeats were found in transcripts of *Sm*PoMuc gr.3 variants of C and IC strains. This suggests that ectopic recombination occurs to generate this genomic level of polymorphism. Different structural elements could explain these frequent recombination events. Genomic repeats (exon 2 and flanking intronic sequences) are conserved within and between all genes of the *Sm*PoMuc family. The level of identity (>93%) shows that these genomic repeats do not evolve independently of each other. The molecular process that leads to homogenization of DNA sequences of a given repetitive family is called “concerted evolution” and occurs in ribosomal genes and in certain protein-coding multigene families, such as those encoding histones or ubiquitin (see [45], for review). This phenomenon was also previously described within several genes (i) encoding proteins containing tandemly repeated domains and (ii) displaying genomic repeats like those of *Sm*PoMuc genes. This is the case for the repetitive part of the single copy gene encoding the coccidioides spherule outer wall glycoprotein (SOWgp). This protein contributes to the virulence of *Coccidioides spp* both by functioning as an adhesion molecule and by modulating the host's immune response [46],[47]. In this SOWgp gene, genomic repeats corresponding to repeated exons and associated introns are also nearly identical and evolve by concerted evolution. This type of evolutionary process can be driven by directional and/or stochastic processes. The two mechanisms that are the principal explanations of concerted evolution in nuclear DNA are gene conversion and unequal crossing over [41]. For unequal crossing over, increases and decreases in repeat number lead to turnover among repeats and, in principle, stochastic fixation of a single repeat type. According to this model, unequal crossing-over commonly occurs in central regions of the array where repeats can mispair, and unique sequences flanking the repetitive array inhibit exchanges in the edge repeats, i.e. the repeats located at the termini. The involvement of unequal crossing-over was shown for the gene encoding SOWgp [48] and support for this view comes from both the higher conservation of repeats in the center of the repetitive array, and the polymorphism in repeat number in SOWgp. The phenomenon of unequal crossing-over probably occurs in members of the *Sm*PoMuc family because our Southern blot results indicate a difference in repeat number between individuals, differences that occur when unequal crossing-over takes place. Nevertheless, unequal crossing-over cannot explain homogenization of genomic repeats containing exon 2 between genes situated on different loci and chromosomes. In addition, we did not find a gradient of conservation between central and edge repeats. We therefore favour the alternative hypothesis of gene conversion (non-reciprocal transfer of information). The molecular mechanism of gene conversion in multigene families is not well understood, nevertheless several findings indicate that cis-acting sequences can influence this phenomenon. One example is provided by the two early chorionic-gene families, *ErA* and *ErB*, of the silk moth *Bombyx mori* [49] which are in close proximity on the same chromosome. The genes of the ErA family exhibit 96% sequence identity, whereas those in the ErB have only 63% sequence identity. Sequence analysis suggested that microsatellite-like simple repeats present in the ErA family, but not in the ErB family, account for the difference in homogenization, because simple sequence repeats can be the sites for initiation of gene conversion [49]. Another well documented example is given by the microsatellite sequences in the human *RNU2* locus that were proposed to play a role in concerted evolution [50]. These observations support the hypothesis that the microsatellites that separate the exon 2 genomic repeats in the *Sm*PoMuc genes are involved in the gene conversion mechanism leading to the concerted evolution of these repeats. Several hypotheses for the mechanism of gene conversion induced by microsatellites have been evoked. These microsatellites composed of purines are polypurine tracts that can adopt a triple helix conformation called H-DNA (see [51] for review). Recently, a major role for this kind of conformation (non-B DNA conformation) in chromosomal rearrangements was proposed. Hotspots of rearrangements occurred invariably at nucleotides abutting or within motifs capable of adopting non-B conformations leading to single or double-stands breaks [52],[53]. The DNA-break repair mechanism could involve invasive DNA replication leading to gene conversion as shown in yeast [54],[55]. These different mechanisms could occur in *Sm*PoMuc genes and explain the similarities observed between the genomic repeats in all members of the multigene family. Nevertheless, this gene conversion phenomenon is restricted to the intronic sequence of these genomic repeats as we have shown that exons are different between clusters. We therefore conclude that conservation of exon differences is due to selection pressure. Interestingly, the same molecular architecture and the same type of evolutionary processes were described for the above-mentioned modular spider silk protein genes. In these genes, repeated structures in the proteins correspond to genomic tandem repeats composed of exons and introns. Once more, in this case genomic repeats are subject to concerted evolution and intron sequences are more homogenized than are the exons that evolve under purifying selection [56]. In *Sm*PoMuc genes, the combination of concerted evolution acting on all repeats and exon difference conservation between clusters (by purifying selection) allows combinatory events that we observed at the genomic level (r1 and r2 exons in the same gene) and in cDNA (r1 and r2 repeats in the same variant) in both strains. The reason is probably ectopic recombination and exon exchange between clusters. A similar phenomenon was described for homologous sequences in somatic plant cells [57].

In addition to the structure of the genes that favour generation of polymorphism on a genomic level, several processes generating further levels of polymorphism occur during the expression of these genes.

First, genes are transcribed in an individual-specific manner. Some individuals express several genes and/or alleles for one group of *Sm*PoMuc and others for two groups that may be different between individuals. This observation raises the question of differential transcriptional regulation of the genes belonging to this multigene family. Future studies will address this question.

Second, we found evidence for various post-transcriptional regulation events. Exhaustive analysis of *Sm*PoMuc cDNAs reveals numerous alternative splicing and aberrant splicing events in the coding region in the 234 residue C-terminal region of the precursor. Alternative splicing events do not change the ORF and lead to shorter proteins. Aberrant splicing also appears frequently and produces a non-sense codon immediately downstream of the splice sites, leading to truncated proteins. As a first approach to investigate the consequence of these events on gene products, we analyzed the predicted glycosylation of these truncated forms of *Sm*PoMuc proteins. An *in silico* approach revealed that these events can have a marked influence on the predicted glycosylation status of *Sm*PoMuc proteins (Table S2) since truncated *Sm*PoMucs are predicted to be more highly glycosylated than are complete proteins. There are many more truncated forms in the IC strain than in the C strain. Our analysis of the global glycosylation status of *Sm*PoMucs by chemical deglycosylation and subsequent western blotting strengthen these predictions of the glycosylation status since molecular weight shifts were larger for IC strain *Sm*PoMucs compared to the C strain. These results support the view that differential splicing events can influence the glycosylation status of *Sm*PoMucs.

In the repeated region of the precursor, a difference in repeat number and repeat combination (r1 and r2 together in the same variant) between *Sm*PoMuc variants is apparent. These variations also influence the predicted glycosylation status of the repeats (see Table S2). This level of polymorphism is probably generated by two mechanisms. The first acts at the genomic level and is related to (i) unequal crossing-over leading to the contraction/expansion of genomic repeat number or (ii) ectopic recombination leading to the combinatory polymorphism found in some variants. But these events that act at the genomic level cannot alone explain the polymorphism observed, particularly the length polymorphism of the TR stretch. Indeed, a maximum of 20 genomic repeats is observed in DNA from both strains and on BACs. Nevertheless, 50% of the *Sm*PoMuc cDNA variants possess more than 20 repetitive units (25–100 repeats). The fact that long repeats in the transcripts are always composed of the same TR type in a given variant suggests that these processes involve alleles (intergenic trans-splicing) or occur in cis (exon repetition). A trans-splicing mechanism was reported for *S. mansoni* [58]–[60] but its function in *S. mansoni* and the absence of a spliced leader sequence in *Sm*PoMuc transcripts suggest that this mechanism is not involved in the phenomenon we observe. Exon repetition was first identified for the rat carnitine octanoyltransferase gene for which two copies of exon 2 were positioned adjacent to one another in some mRNAs while the genomic sequence contained only a single copy [61]. This mechanism has been further studied [62],[63] and the intervention of complementary intron sequences has been hypothesized [64]. Since we detected two complementary sequences of 13 and 12 nucleotides (Figure 3), respectively, in intronic sequences flanking exon 2 (data not shown), this hypothesis can be proposed for *Sm*PoMuc genes. In addition, the presence of hammerhead ribozymes in all *Sm*PoMuc genes is intriguing. *S. mansoni* hammerhead ribozymes were extensively studied and shown to catalyze cleavage [35] and ligation [36] of transcripts *in vitro*. Their *in vivo* function is unknown but we can hypothesize that *S. mansoni* hammerhead ribozymes play a role in *Sm*PoMuc transcript processing. This hypothesis is strengthened by a structural characteristic revealed by the analysis of *Sm*PoMuc genes and transcripts corresponding to gr.2. Group 2 *Sm*PoMuc genes are the only ones that possess the G12 of the catalytic core that was shown to be essential for ribozyme activity [39]. The corresponding transcripts more frequently show exon repetition than others (see Table S1).

**Figure 11:**
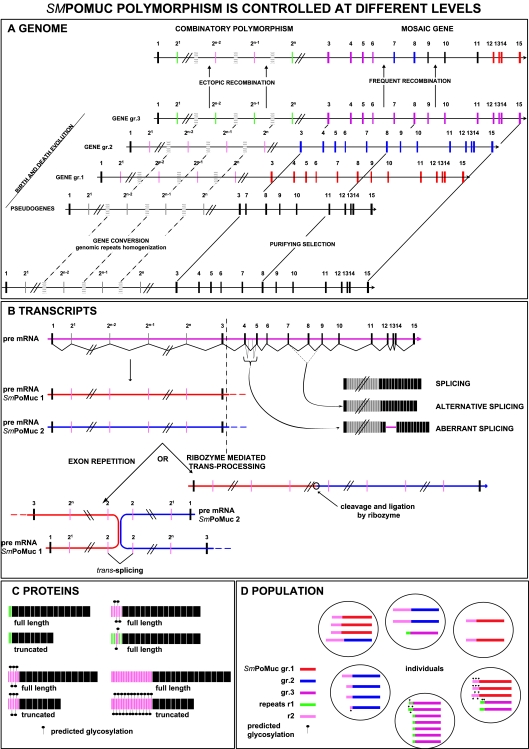
Controlled chaos of *Sm*PoMuc polymorphism. *Sm*PoMuc polymorphism is controlled at the genomic (A), transcript (B), protein (C) and population (D) levels.

All the data we present here for the *Sm*PoMuc multigene family show that gene structure, genomic organisation, recombination events and different regulation mechanisms during their expression allow the generation of a remarkably high degree of polymorphism from a limited set of genes (see Figure 11 for a schematic representation). This characteristic is unique for this model compared to the expression of polymorphic molecular variants in other parasites. Indeed, in all previously described cases the molecular variants are synthesized from a large set of genes belonging to a multigene family. An example is the case of *Trypanosoma cruzi* surface mucins (see [65] for review) that contribute to parasite protection and to the establishment of a persistent infection. The multigene family encoding these proteins comprises 850 genes covering 1% of the parasite genome. Other relevant gene families include the *vsg* or the *var* family responsible for antigenic variation of *Trypanosoma brucei* (see [66],[67]) or *P. falciparum* [68],[69]. *T. brucei* has >1000 *vsg* genes and pseudogenes and the genome project of *P. falciporum* has identified 59 intact *var* genes.

In the present study, we provide evidence for several events acting at the genomic level and during expression leading to an extraordinary level of *Sm*PoMucs polymorphism at inter and intra strain levels. Population studies have shown that success or failure of *B. glabrata/S. mansoni* infection depends on the matched or mismatched status of the host and parasite phenotypes [15]. Interestingly, recent studies have demonstrated that the previously characterized family of *Biomphalaria* FREPs undergo processes of recombination diversification leading to the concomitant existence of a great diversity of FREPs within a single individual [13]. Although their function has not yet been totally clarified, evidence suggests that FREPs are capable of binding molecules of foreign origin such as *Echinostoma paraensei* excretory-secretory products [16] via their carbohydrate recognition domain. The exposure of host carbohydrate-binding molecules such as FREPs, to their *S. mansoni* carbohydrate ligands could determine the matched or mismatched status of a given *B. glabrata-S. mansoni* combination. In this context, *Sm*PoMucs from *S. mansoni* are very promising candidates. These molecules are different between compatible and incompatible strains and are secreted and glycosylated [17],[18]. In addition, parasite-derived mucins or mucin-like molecules have been extensively described in different protozoan and helminth parasites. They have roles in host recognition, penetration, adhesion and invasion of host cells, immunoprotection, immunomodulation and in the avoidance of host immune processes [70],[71]. Among these molecules in parasites, the mucin-like proteins of *T. cruzi* share numerous characteristics with *Sm*PoMuc (see [65] for review), such as their structure, a high level of glycosylation and polymorphism. A major difference is that these molecules, encoded by the TcMUC I and II genes, like other surface components of parasitic protozoa, are typically anchored to the outer phospholipid layer of the plasma membrane by GPI. Nevertheless, secreted mucins have been characterized in helminths such as the infective larvae of the parasitic nematode *Toxocara canis* [72]. These secreted mucins might simply create an immunological smoke-screen by trapping antigen-antibody complexes away from the parasite [73]. An attractive hypothesis is that *Sm*PoMucs create an immunological smoke-screen able to block pattern recognition receptors (like FREPs), thus avoiding recognition and the host immune reaction. Finally, we show here that *Sm*PoMucs display the level of polymorphism we expect for key determinants of the compatibility polymorphism in play between *B. glabrata* and *S. mansoni*. We show that this high level of polymorphism has a consequence in the glycosylation status of *Sm*PoMuc. All these data, taken together, strengthen our hypothesis that *Sm*PoMucs are key determinants of *S. mansoni/B. glabrata* compatibility polymorphism.

## Supporting Information

Table S1 cDNA variants obtained for 11 individuals from both Compatible and Incompatible strains. ^a^Strain Compatible (C) or Incompatible (IC) and individual number (1–11). ^b^ref-(x) = variant used as reference for mutation identification, x refers to *Sm*PoMuc group. ^c^TR = Tandem Repeats contained in each variant. - number and type of TR is indicated. - °(x) Synonymous mutation (SM) present in the x^th^ repeat of the stretch. - *(x) Non synonymous mutation (NSM) present in the x^th^ repeat of the stretch. ^d^exon characteristics of each variant: - exon presence or absence is annotated by x or in grey, respectively. - green, blue, orange and red exons contain SM, NSM, SM+NSM and STOP codon, respectively. - //+ indicates partial intron inclusion. - //− indicates partial exon exclusion. ^e^splicing events: - AS = Alternative Splicing with conservation of ORF. - AbS (x) = Aberrant Splicing; x refer to AbS type described in Figure 9.
(447 KB PDF)

Table S2 Glycosylation prediction on Tandem Repeats from Compatible and Incompatible cDNA variants. ^a^Strain Compatible (C) or Incompatible (IC) and individual number (1–11). ^b^Variant Id. = Variant Identification (Individual/*Sm*PoMuc group/associated tandem repeat stretch). *Sm*PoMuc groups are color coded: gr1, 2, and 3.1 in blue, yellow and purple, respectively. ^c^Splicing variant are indicated by a red box: - AS = Alternative Splicing with conservation of ORF. - AbS (x) = Aberrant Splicing; x refer to AbS type described in Figure 9. ^d^TR = Tandem Repeats contained in each variant. - number and type of TR is indicated. - °(x) Synonymous mutation (SM) present in the x^th^ repeat of the stretch. - *(x) Non synonymous mutation (NSM) present in the x^th^ repeat of the stretch. ^e^Glycosylation site prediction in tandem repeats from the different variants: - repeats containing no prediction site are represented as white boxes. - r1 and r2 repeats containing 1 prediction site on T9 are represented as dark grey boxes. - r2 repeats containing 2 prediction sites on S5 and T9 are represented as dotted boxes.
(389 KB PDF)
