# Supplementary material for: Controlled Chaos of Polymorphic Mucins in a Metazoan Parasite (Schistosoma mansoni) Interacting with Its Invertebrate Host (Biomphalaria glabrata)
Source: PLoS Negl Trop Dis. 2008 Nov 11;2(11):e330. doi: 10.1371/journal.pntd.0000330 (PMC2576457; doi:10.1371/journal.pntd.0000330)
Supplement: Table S1 — cDNA variants obtained for 11 individuals from both Compatible and Incompatible strains. aStrain Compatible (C) or Incompatible (IC) and individual number (1–11). bref-(x) = variant used as reference for mutation identification, x refers to SmPoMuc group. cTR = Tandem Repeats contained in each variant. - number and type of TR is indicated. - °(x) Synonymous mutation (SM) present in the xth repeat of the stretch. - *(x) Non synonymous mutation (NSM) present in the xth repeat of the stretch. dexon characteristics of each variant: - exon presence or absence is annotated by x or in grey, respectively. - green, blue, orange and red exons contain SM, NSM, SM+NSM and STOP codon, respectively. - //+ indicates partial intron inclusion. - //− indicates partial exon exclusion. esplicing events: - AS = Alternative Splicing with conservation of ORF. - AbS (x) = Aberrant Splicing; x refer to AbS type described in Figure 9. (447 KB PDF) [file pntd.0000330.s001.pdf]

Table S1: cDNA variants obtained for 11 individuals from both Compatible and Incompatible strains

Compatible strain																
ind[a]	*Sm* PoMuc[b] group	TR[c]	exon[d]													splicing[e]
			3	4	5	6	7	8	9	10	11	12	13	14	15	
C - 1	3.1	2r1	x	x	x		x	x	x			x	x	x	x	AS
	ref-3.1	2r1	x	x	x	x	x	x	x	x	x	x	x	x	x	
		r1									x	x	x	x	x	AS
		r1									x	x	x	x	x	AS
		r1'										x	x	x	x	AS
		r1'										x	x	x	x	AS
	2	7r2										x	x	x	x	AS
		7r2[°2-4]										x	x	x	x	AS
C - 2	3.2	r1	x				x	x	x	x	x	x	x	x	x	AS
C -3	2	≈ 45r2	x	x	x	x	x	// -	STOP	x	x	x	x	x	x	AbS (1)
		≈ 40r2	x	x	x	x	x	// -	STOP	x	x	x	x	x	x	AbS (1)
		≈ 25r2	x	x	x	x	x	// -	STOP	x	x	x	x	x	x	AbS (1)
		11r2[°2]	x	x	x	x	x	x	x	x	x	x	x	x	x	
C - 4	3.1	9r1'-r1-r1'	x	x	x	x	x	x	x	x	x	x	x	x	x	
	2	≈ 100r2	x	x	x	x	x		x	x	x	x	x	x	x	AS
		≈ 100r2[*3]	x	x	x	x	x		x	x	x	x	x	x	x	AS
C - 5	3.1	2r1'	x	x	x	x	x	x	x	x	x	x	x	x	x	
		2r1'	x	x	x	x	x	x	x	x	x	x	x	x	x	
		2r1'	x	x	x	x	x	x	x	x	x	x	x	x	x	
		2r1'[*2]	x	x	x	x	x	x	x	x	x	x	x	x	x	
C - 6	1	≈ 40r2	x	x	x	x	x	x	x	x	x	x	x	x	x	
		≈ 40r2	x	x	x	x	x	x	x	x	x	x	x	x	x	
		≈ 40r2	x	x	x	x	x	x	x	x	x	x	x	x	x	
	2	≈ 80r2	x	x	x	x	x	x	x	x	x	x	x	x	x	
C - 7	2	≈ 25r2	x	x	x	x	x	x	x	x	x	x	x	x	x	
	ref-2	≈ 25r2	x	x	x	x	x	x	x	x	x	x	x	x	x	
		≈ 25r2	x	x	x	x	x	x	x	x	x	x	x	x	x	
		≈ 25r2	x	x	x	x	x	x	x	x	x	x	x	x	x	
		≈ 25r2	x	x	x	x	x	x	x	x	x	x	x	x	x	
		≈ 30r2	x	x	x	x	x	x	x	x	x	x	x	x	x	
C - 8	3.1	2r1-2r2[*2]-r1-r1'	x	x	x	x	x	x	x	x	x	x	x	x	x	
C - 9	3.1	2r1'	x	x	x	x	x	x	x	x	x	x	x	x	x	
		2r1'[*2]	x	x	x	x	x	x	x	x	x	x	x	x	x	
		4r1-r1'-4r1	x	x	x	x	x	x	x	x	x	x	x	x	x	
C - 10	1	≈ 40r2	x	x	x	x	x	x	x	x	x	x	x	x	x	
		≈ 40r2	x	x	x	x	x	x	x	x	x	x	x	x	x	
C - 11	3.1	4r1[*2]-r1'-4r1	x	x	x	x	x	x	x	x	x	x	x	x	x	
		9r1'-r1-r1'	x	x	x	x	x	x	x	x	x	x	x	x	x	
	1	18r2	x	x	x	x	x	x	x	x	x	x	x	x	x	
		15r2	x	x	x	x	x	x	x	x	x	x	x	x	x	
	ref-1	15r2	x	x	x	x	x	x	x	x	x	x	x	x	x	

Incompatible strain

ind[a]	*Sm* PoMuc[b] group	TR[c]	exon[d] 3	4	5	6	7	8	9	10	11	12	13	14	15	splicing[e]
IC - 1	3.1	9r1*2	x	x	x	x	x	x	x	x	x	x	x	x	x	
		2r1-r2-r2*-r1'	x	x	x	x	x	x	x	x	x	x	x	x	x	
		2r1	x	x	x	x	x	x	x	x	x	x	x	x	x	
		2r1	x	x	x	x	x	x	x	x	x	x	x	x	x	
		2r1	x	x	x	x	x	x	x	x	x	x	x	x	x	
		2r1	x	x	x	x	x	x	x	x	x	x	x	x	x	
		r1-r1'	x	x	x	x	x	x	x		x	x	x	x	x	AS
		r1°-r1'°	x	x	x	x	x	x	x		x	x	x	x	x	AS
		r1-r1'*	x	x	x	x	x	x	x		x	x	x	x	x	AS
IC - 2	2	≈ 25r2*	x	x	x	x	x	// -	STOP	x	x	x	x	x	x	AbS (1)
		≈ 25r2	x	x	x	x	x	x	x	x	x	x	x	x	x	
		15r2	x	x	x	x	x	// -	STOP	x	x	x	x	x	x	AbS (1)
		6r2	x	x	x	x	x	x	x	x	x	x	x	x	x	
		4r2		// -	STOP	x	x	// -	x	x	x	x	x	x	x	AbS (3)
	3.1	r1-2r2°2-r1-r1'	x	x	x	x	x	x	x	x	x	x	x	x	x	
		r1-2r2-r1-r1'	x	x	x	x	x	x	x	x	x	x	x	x	x	
		r1-2r2-r1-r1'	x	x	x	x	x	x	x	x	x	x	x	x	x	
		2r1-r1'	x	x	x	x	x	x	x	x	x	x	x	x	x	
IC - 3	2	≈ 40r2	x	x	x	x	x	x	x	x	x	x	x	x	x	
		≈ 30r2	x	x	x	x	x	x	x	x	x	x	x	x	x	
		≈ 25r2	x	x	x	x	x	x	x	x	x	x	x	x	x	
		4r2	x	x	x	x	x	x	x	x	x	x	x	x	x	
IC - 4	2	11r2*11	x	x	x	x	x	// -	STOP	x	x	x	x	x	x	AbS (1)
		13r2°13	x	x	x	x	x	// -	STOP	x	x	x	x	x	x	AbS (1)
		≈ 25r2	x	x	x		x	// -	STOP	x	x	x	x	x	x	AbS (1)
		≈ 30r2°n-1	x	x	x	x	x	// -	STOP	x	x	x	x	x	x	AbS (1)
		10r2	x	x	x	x	x	x	x	x	x	x	x	x	x	
IC - 5	3.1	10r1 //+ STOP	x	x	x	x	x	x	x	x	x	x	x	x	x	AbS (4)
		12r1*7	x	//+ STOP		x	x	x	x	x	x	x	x	x	x	AbS (6)
		11r1*10 //+	x	//+ STOP		x	x	x	x	x	x	x	x	x	x	AbS (5)
		11r1*3	x	x	x	x	x	x	x	x	x	x	x	x	x	
	ref-3.1	11r1	x	x	x	x	x	x	x	x	x	x	x	x	x	
		8r1	x	x	x	x	x	x	x	x	x	x	x	x	x	
		2r1-2r2-r1-r1'	x	x	x	x	x	x	x	x	x	x	x	x	x	
		2r1-2r2-r1-r1'	x	x	x	x	x	x	x	x	x	x	x	x	x	
	2	r2	x	x	x	x	x	x	x	x	x	x	x	x	x	
		10r2*10						x	x	x	x	x	x	x	x	AS
IC - 6	2	≈ 25r2°n-1	x	x	x	x	x	// -	STOP	x	x	x	x	x	x	AbS (1)
		≈ 25r2*7	x	x	x	x	x	// -	STOP	x	x	x	x	x	x	AbS (1)
	ref-2	≈ 25r2	x	x	x	x	x	x	x	x	x	x	x	x	x	
		≈ 25r2	x	x	x	x	x	// -	STOP	x	x	x	x	x	x	AbS (1)
		15r2	x	x	x	x	x	// -	STOP	x	x	x	x	x	x	AbS (1)
IC - 7	3.1	2r1	x	x	x	x	x	x	x	x	x	x	x	x	x	
		2r1	x	x	x	x	x	x	x	x	x	x	x	x	x	

		3r1	x	x	x	x	x	x	x	x	x	x	x	x	x	
		2r1-2r2-r1-r1'	x	x	x	x	x	x	x	x	x	x	x	x	x	
	2	≈ 30r2	x	x	x	x	x	// -	STOP	x	x	x	x	x	x	AbS (1)
		≈ 30r2	x	x	x	x	x		x	x	x	x	x	x	x	AS
		≈ 35r2 [*n]	x	x	x	x	x	// -	STOP	x	x	x	x	x	x	AbS (1)
		18r2	x	x	x	x	x	// -	STOP	x	x	x	x	x	x	AbS (1)
IC - 8	2	8r2	x	x	x	x	x	// -	STOP	x	x	x	x	x	x	AbS (1)
		8r2	x	x	x	x	x	// -	STOP	x	x	x	x	x	x	AbS (1)
		8r2	x	x	x	x	x	// -	STOP	x	x	x	x	x	x	AbS (1)
		7r2	x	x	x	x	x	// -	STOP	x	x	x	x	x	x	AbS (1)
		r2	x	x	x	x	x	// -	STOP	x	x	x	x	x	x	AbS (1)
	3.1	2r1-2r2-r1-r1'	x	x	x	x	x	x	x	x	x	x	x	x	x	
		5r1	x	x	x	x	x	x	x	x	x	x	x	x	x	
IC - 9	2	≈ 25r2	x	x	x	x	x		x	x	x	x	x	x	x	AS
		5r2	x	x	x	x	x	// -	STOP	x	x	x	x	x	x	AbS (1)
		3r2	x	x	x	x	x		x	x	x	x	x	x	x	AS
		9r2 [*2]	x	x	x	x	x	// -	STOP	x	x	x	x	x	x	AbS (1)
		7r2	x	x	x	x	x	x	x	x	x	x	x	x	x	
		2r2	x	x	x	x	x		x	x	x	x	x	x	x	AS
		15r2	x	x	x	x	x	// -	STOP	x	x	x	x	x	x	AbS (1)
		14r2	x	x	x	x	x	// -	STOP	x	x	x	x	x	x	AbS (1)
	3.1	4r1 [°4]	x	x	x	x	x	x	x	x	x	x	x	x	x	
		r1-r1 [*]	x	x	x	x	x	x	x	x	x	x	x	x	x	
IC - 10	2	≈ 25r2	x	x	x	x	x	x	x	x	x	x	x	x	x	
		≈ 30r2	x	x	x	x	x	x	x	x	x	x	x	x	x	
		≈ 30r2	x	x	x	x	x	x	x	x	x	x	x	x	x	
		≈ 30r2	x	x	x	x	x		x	x	x	x	x	x	x	AS
		15r2 [*14]	x	x	x	x	x		x	x	x	x	x	x	x	AS
	3.1	2r1-2r2-r1-r1'	x	x	x	x	x	x	x	x	x	x	x	x	x	
		11r1 [°11]	x	x	x	x	x	x	x	x	x	x	x	x	x	
		11r1	x	x	x	x	x	x	x	x	x	x	x	x	x	
		11r1 [°1]	x	x	x	x	x	x	x	x	x	x	x	x	x	
IC - 11	2	≈ 20r2 [*n-1]	x	x	x	x	x	// -	STOP	x	x	x	x	x	x	AbS (1)
		≈ 30r2	x	x	x	x	x	// -	STOP	x	x	x	x	x	x	AbS (1)
		≈ 30r2	x	x	x	x	x	x	x	x	x	x	x	x	x	
		≈ 30r2 [°1-3]	x	x	x	x	x	// -	STOP	x	x	x	x	x	x	AbS (1)
		≈ 30r2	x	x	x	x	x	// -	STOP	x	x	x	x	x	x	AbS (1)
		7r2	x	// -	STOP	x	x	x	x	x	x	x	x	x	x	AbS (2)
		10r2	x	x	x	x	x	// -	STOP	x	x	x	x	x	x	AbS (1)
		13r2	x	x	x	x	x	// -	STOP	x	x	x	x	x	x	AbS (1)
