# Supplementary material for: Controlled Chaos of Polymorphic Mucins in a Metazoan Parasite (Schistosoma mansoni) Interacting with Its Invertebrate Host (Biomphalaria glabrata)
Source: PLoS Negl Trop Dis. 2008 Nov 11;2(11):e330. doi: 10.1371/journal.pntd.0000330 (PMC2576457; doi:10.1371/journal.pntd.0000330)
Supplement: Table S2 — Glycosylation prediction on Tandem Repeats from Compatible and Incompatible cDNA variants. aStrain Compatible (C) or Incompatible (IC) and individual number (1–11). bVariant Id. = Variant Identification (Individual/SmPoMuc group/associated tandem repeat stretch). SmPoMuc groups are color coded: gr1, 2, and 3.1 in blue, yellow and purple, respectively. cSplicing variant are indicated by a red box: - AS = Alternative Splicing with conservation of ORF. - AbS (x) = Aberrant Splicing; x refer to AbS type described in Figure 9. dTR = Tandem Repeats contained in each variant. - number and type of TR is indicated. - °(x) Synonymous mutation (SM) present in the xth repeat of the stretch. - *(x) Non synonymous mutation (NSM) present in the xth repeat of the stretch. eGlycosylation site prediction in tandem repeats from the different variants: - repeats containing no prediction site are represented as white boxes. - r1 and r2 repeats containing 1 prediction site on T9 are represented as dark grey boxes. - r2 repeats containing 2 prediction sites on S5 and T9 are represented as dotted boxes. (389 KB PDF) [file pntd.0000330.s002.pdf]

Table S2: Glycosylation prediction on Tandem Repeats from Compatible and Incompatible cDNA variants

Compatible strain

ind[a]	Variant id.[b]	splicing[c]	TR[d]	glycosylation prediction in TR[e] (1–35)
C - 1	C1/3.1/2r1.1	NO	2r1	
	C1/3.1/2r1.2	AS	2r1	
	C1/3.1/r1.1	AS	r1	
	C1/3.1/r1.2	AS	r1	
	C1/3.1/r1'.1	AS	r1'	
	C1/3.1/r1'.2	AS	r1'	
	C1/2/7r2.1	AS	7r2	
	C1/2/7r2.2	AS	7r2°2-4	
C - 2	C2/3.2/r1	AS	r1	
C -3	C3/2/45r2	AbS (1)	≈ 45r2	
	C3/2/40r2	AbS (1)	≈ 40r2	
	C3/2/25r2	AbS (1)	≈ 25r2	
	C3/2/11r2	NO	11r2°2	
C - 4	C4/3.1/9r1'-r1-r1'	NO	9r1'-r1-r1'	
	C4/2/100r2.1	AS	≈ 100r2	
	C4/2/100r2.2	AS	≈ 100r2*3	
C - 5	C5/3.1/2r1'.1	NO	2r1'	
	C5/3.1/2r1'.2	NO	2r1'	
	C5/3.1/2r1'.3	NO	2r1'	
	C5/3.1/2r1'.4	NO	2r1'*2	
C - 6	C6/1/40r2.1	NO	≈ 40r2	
	C6/1/40r2.2	NO	≈ 40r2	
	C6/1/40r2.3	NO	≈ 40r2	
	C6/2/80r2	NO	≈ 80r2	
C - 7	C7/2/25r2.1	NO	≈ 25r2	
	C7/2/25r2.2	NO	≈ 25r2	
	C7/2/25r2.3	NO	≈ 25r2	
	C7/2/25r2.4	NO	≈ 25r2	
	C7/2/25r2.5	NO	≈ 25r2	
	C7/2/30r2	NO	≈ 30r2	
C - 8	C8/3.1/2r1-2r2-r1-r1'	NO	2r1-2r2*2-r1-r1'	
C - 9	C9/3.1/2r1'.1	NO	2r1'	
	C9/3.1/2r1'.2	NO	2r1'*2	
	C9/3.1/4r1-r1'-4r1	NO	4r1-r1'-4r1	
C - 10	C10/1/40r2.1	NO	≈ 40r2	
	C10/1/40r2.2	NO	≈ 40r2	
C - 11	C11/3.1/4r1-r1'-4r1	NO	4r1*2-r1'-4r1	
	C11/3.1/9r1'-r1-r1'	NO	9r1'-r1-r1'	
	C11/1/18r2	NO	18r2	
	C11/1/15r2.1	NO	15r2	
	C11/1/15r2.2	NO	15r2	

Incompatible strain

ind[a]	Variant id.[b]	splicing[c]	TR[d]	glycosylation prediction in TR[e] (1–35)
IC - 1	IC1/3.1/9r1	NO	9r1*2	
	IC1/3.1/2r1-2r2-r1'	NO	2r1-r2-r2*-r1'	
	IC1/3.1/2r1.1	NO	2r1	
	IC1/3.1/2r1.2	NO	2r1	
	IC1/3.1/2r1.3	NO	2r1	
	IC1/3.1/2r1.4	NO	2r1	
	IC1/3.1/r1-r1'.1	AS	r1-r1'	
	IC1/3.1/r1-r1'.2	AS	r1°-r1'°	
	IC1/3.1/r1-r1'.3	AS	r1-r1'*	
IC - 2	IC2/2/25r2.1	AbS (1)	≈ 25r2*	
	IC2/2/25r2.2	NO	≈ 25r2	
	IC2/2/15r2	AbS (1)	15r2	
	IC2/2/6r2	NO	6r2	
	IC2/2/4r2	AbS (3)	4r2	
	IC2/3.1/r1-2r2-r1-r1'.1	NO	r1-2r2°2-r1-r1'	
	IC2/3.1/r1-2r2-r1-r1'.2	NO	r1-2r2-r1-r1'	
	IC2/3.1/r1-2r2-r1-r1'.3	NO	r1-2r2-r1-r1'	
	IC2/3.1/2r1-r1'	NO	2r1-r1'	
IC - 3	IC3/2/40r2	NO	≈ 40r2	
	IC3/2/30r2	NO	≈ 30r2	
	IC3/2/25r2	NO	≈ 25r2	

	IC3/2/4r2	NO	4r2
IC - 4	IC4/2/11r2	AbS (1)	11r2^{*11}
	IC4/2/13r2	AbS (1)	13r2$^{\circ 13}$
	IC4/2/25r2	AbS (1)	≈ 25r2
	IC4/2/30r2	AbS (1)	≈ 30r2$^{\circ n-1}$
	IC4/2/10r2	NO	10r2
IC - 5	IC5/3.1/10r1	AbS (4)	10r1 //STOP
	IC5/3.1/12r1	AbS (6)	12r1^{*7}
	IC5/3.1/11r1.1	AbS (5)	11r1^{*10} //
	IC5/3.1/11r1.2	NO	11r1^{*3}
	IC5/3.1/11r1.3	NO	11r1
	IC5/3.1/8r1	NO	8r1
	IC5/3.1/2r1-2r2-r1-r1'.1	NO	2r1-2r2-r1-r1'
	IC5/3.1/2r1-2r2-r1-r1'.2	NO	2r1-2r2-r1-r1'
	IC5/2/1r2	NO	r2
	IC5/2/10r2	AS	10r2^{*10}
IC - 6	IC6/2/25r2.1	AbS (1)	≈ 25r2$^{\circ n-1}$
	IC6/2/25r2.2	AbS (1)	≈ 25r2^{*7}
	IC6/2/25r2.3	NO	≈ 25r2
	IC6/2/25r2.4	AbS (1)	≈ 25r2
	IC6/2/15r2	AbS (1)	15r2
IC - 7	IC7/3.1/2r1.1	NO	2r1
	IC7/3.1/2r1.2	NO	2r1
	IC7/3.1/3r1	NO	3r1
	IC7/3.1/2r1-2r2-r1-r1'	NO	2r1-2r2-r1-r1'
	IC7/2/30r2.1	AbS (1)	≈ 30r2
	IC7/2/30r2.2	AS	≈ 30r2
	IC7/2/35r2	AbS (1)	≈ 35r2^{*n}
	IC7/2/18r2	AbS (1)	18r2
IC - 8	IC8/2/8r2.1	AbS (1)	8r2
	IC8/2/8r2.2	AbS (1)	8r2
	IC8/2/8r2.3	AbS (1)	8r2
	IC8/2/7r2	AbS (1)	7r2
	IC8/2/r2	AbS (1)	r2
	IC8/3.1/2r1-2r2-r1-r1'	NO	2r1-2r2-r1-r1'
	IC8/3.1/5r1	NO	5r1
IC - 9	IC9/2/25r2	AS	≈ 25r2
	IC9/2/5r2	AbS (1)	5r2
	IC9/2/3r2	AS	3r2
	IC9/2/9r2	AbS (1)	9r2^{*2}
	IC9/2/7r2	NO	7r2
	IC9/2/2r2	AS	2r2
	IC9/2/15r2	AbS (1)	15r2
	IC9/2/14r2	AbS (1)	14r2
	IC9/3.1/4r1	NO	4r1$^{\circ 4}$
	IC9/3.1/2r1	NO	r1-r1*
IC - 10	IC10/2/25r2	NO	≈ 25r2
	IC10/2/30r2.1	NO	≈ 30r2
	IC10/2/30r2.2	NO	≈ 30r2
	IC10/2/30r2.3	AS	≈ 30r2
	IC10/2/15r2	AS	15r2^{*14}
	IC10/3.1/2r1-2r2-r1-r1'	NO	2r1-2r2-r1-r1'
	IC10/3.1/11r1.1	NO	11r1$^{\circ 11}$
	IC10/3.1/11r1.2	NO	11r1
	IC10/3.1/11r1.3	NO	11r1$^{\circ 1}$
IC - 11	IC11/2/20r2	AbS (1)	≈ 20r2^{*n-1}
	IC11/2/30r2.1	AbS (1)	≈ 30r2
	IC11/2/30r2.2	NO	≈ 30r2
	IC11/2/30r2.3	AbS (1)	≈ 30r2$^{\circ 1-3}$
	IC11/2/30r2.4	AbS (1)	≈ 30r2
	IC11/2/7r2	AbS (2)	7r2
	IC11/2/10r2	AbS (1)	10r2
	IC11/2/13r2	AbS (1)	13r2
